# Transgenerational Self-Reconstruction of Disrupted Chromatin Organization After Exposure To An Environmental Stressor in Mice

**DOI:** 10.1038/s41598-019-49440-2

**Published:** 2019-09-10

**Authors:** Carlos Diaz-Castillo, Raquel Chamorro-Garcia, Toshi Shioda, Bruce Blumberg

**Affiliations:** 1 Department of Developmental and Cell Biology, University of California, Irvine, 2011 Biological Sciences 3, Irvine, 92697-2300 USA; 2 Present Address: Department of Microbiology and Environmental Toxicology, University of California, Santa Cruz, 1156 High Street, Santa Cruz, CA 95064 USA; 3 Center for Cancer Research, Massachusetts General Hospital, Bldg 149, 13th Street, Charlestown, MA 02129 USA; 4 Department of Pharmaceutical Sciences, University of California, Irvine, Irvine, 92697-2300 USA

**Keywords:** Transcriptomics, Obesity

## Abstract

Exposure to environmental stressors is known to increase disease susceptibility in unexposed descendants in the absence of detectable genetic mutations. The mechanisms mediating environmentally-induced transgenerational disease susceptibility are poorly understood. We showed that great-great-grandsons of female mice exposed to tributyltin (TBT) throughout pregnancy and lactation were predisposed to obesity due to altered chromatin organization that subsequently biased DNA methylation and gene expression. Here we analyzed DNA methylomes and transcriptomes from tissues of animals ancestrally exposed to TBT spanning generations, sexes, ontogeny, and cell differentiation state. We found that TBT elicited concerted alterations in the expression of “*chromatin organization*” genes and inferred that TBT-disrupted chromatin organization might be able to self-reconstruct transgenerationally. We also found that the location of “*chromatin organization*” and “*metabolic*” genes is biased similarly in mouse and human genomes, suggesting that exposure to environmental stressors in different species could elicit similar phenotypic effects via self-reconstruction of disrupted chromatin organization.

## Introduction

There is currently an obesity pandemic; 39.8% of the US adult population is clinically obese (body mass index >30 kg/m^2^)^1^ adding substantially to healthcare costs^2^. Identifying the full spectrum of factors that may predispose to obesity is key for its treatment and prevention. Obesogens are environmental stressors that promote adiposity by altering fat cell number, size, homeostasis and/or hormonal regulation of metabolism, appetite, and satiety^3^. Although there is a paucity of information regarding human exposure to obesogens, an important recent study revealed that individuals with the highest levels of perfluorinated compounds in plasma had lower resting metabolic rates and regained lost weight more quickly after cessation of a weight-loss program^4^. A number of environmental chemicals were shown to exert obesogenic effects in animal models, in directly exposed animals and in some cases on their unexposed descendants. However, the mechanisms through which most of these chemicals act remain poorly understood^5,6^.

One obesogen that has been extensively studied in our lab and elsewhere is tributyltin (TBT). TBT was used as antifouling agent with strong biocidal activity, as a fungicide, wood preservative and occurs as a contaminant in vinyl plastics^7–10^. TBT was found in human blood at concentrations between 3 nM-260 nM, in house dust, and continues to contaminate sea food, suggesting that human exposure occurs via inhalation and ingestion^11–16^. One study from a Finnish cohort of newborn boys showed a positive association between levels of TBT in placentas and increased body weight in the first 18 months of life^17^. TBT activates the peroxisome proliferator-activated receptor gamma (PPARγ) and its heterodimeric partner, the retinoid X receptor (RXR), which together regulate adipogenic commitment and differentiation^18–20^. We showed that prenatal TBT exposure leads to increased fat storage and the commitment of multipotent mesenchymal stem cells (MSCs) to the adipogenic lineage in mice^19–24^. TBT exposure in adult rats resulted in altered hypothalamic-pituitary-gonadal axis and increased fat storage in the female gonads^25–28^. Animals ancestrally exposed to TBT showed increased white adipose tissue (WAT) mass, white adipocyte number and size, increased commitment of MSCs to the adipocyte lineage, and significant hepatic steatosis^22^. We reproduced these findings in a separate transgenerational experiment and showed that F4 male descendants of TBT exposed dams had increased plasma leptin levels and accumulated significantly more fat than control animals when their diets where switched from a standard diet (13.2% Kcal from fat) to a diet with a slightly higher content of fat (21.6% Kcal from fat). Moreover, their ability to mobilize stored fat during fasting was impaired^21^.

To gain a deeper understanding of the molecular mechanisms underlying TBT-dependent transgenerational obesity, we performed integrative analyses of DNA methylomes and transcriptomes of F4 male gonadal WAT (gWAT) and F3/F4 sperm chromatin accessibility from animals ancestrally exposed to TBT and controls^21^. The initial focus of these analyses was on potential associations between alterations in the DNA methylation state of promoter regions and the expression of genes relevant to metabolic disorders. However, the number of DNA methylation alterations located in promoter regions was negligible and had no obvious relationship to alterations in gene expression. Next, we followed a less gene-centric approach to identify potential larger-scale DNA methylation and transcriptomic alterations. We identified regions in gWAT DNA that were punctuated by either hypo- or hyper-methylated blocks of DNA in TBT samples when compared to controls and denoted these as iso-directional differentially methylated blocks (isoDMBs)^21^. We observed that alterations in TBT-dependent DNA methylome, transcriptome, and chromatin accessibility were distributed dichotomously with respect to genomic regions of biased base composition. GC-enriched genomic regions were preferentially associated with hypomethylated isoDMBs and increased expression of metabolically-relevant genes in gWAT; these regions were less accessible (as measured by ATAC-seq) in F3/F4 sperm. In contrast, AT-enriched regions were preferentially associated with hypermethylated isoDMBs in gWAT and were more accessible in F3/F4 sperm. We proposed that ancestral exposure to the environmental stressor TBT resulted in disruptions of higher order chromatin organization that (i) subsequently biased genomic traits such as DNA methylation, chromatin accessibility or gene expression, (ii) was propagated through development and across generations, and (iii) predisposed descendants of exposed individuals to metabolic disorders^21^.

**Figure 1:**
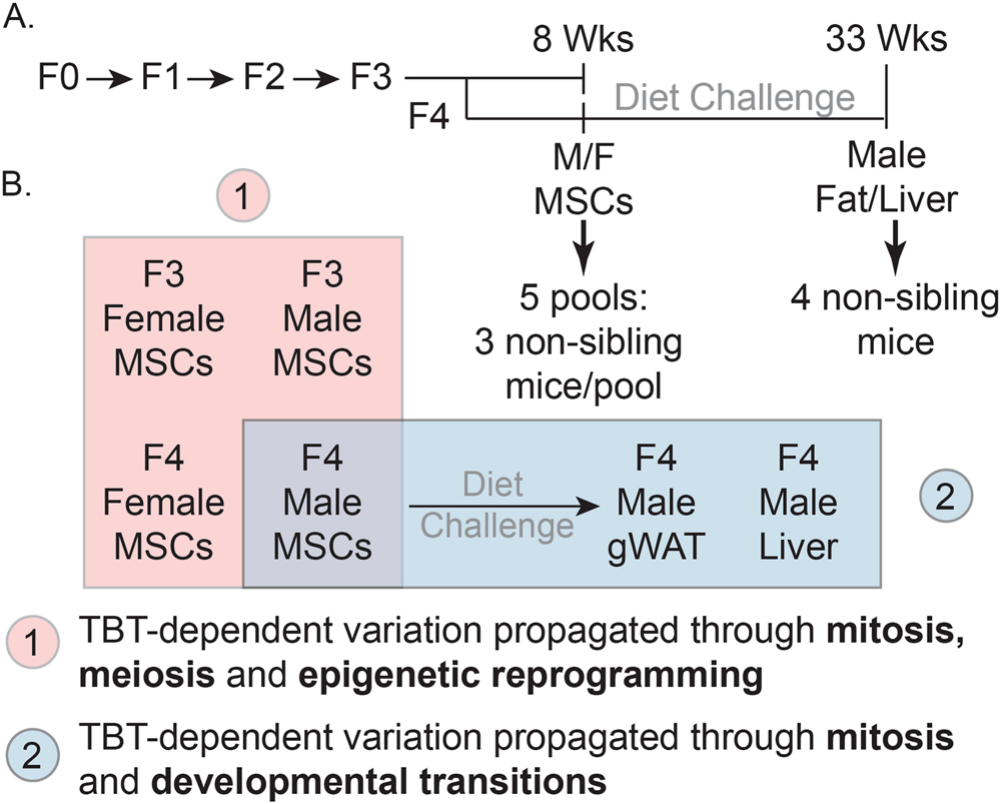
Experimental and analytical design for the study of the intergenerational and developmental dynamics of TBT-dependent variation of DNA methylomes and transcriptomes. (**A**) Experimental design of a TBT-based transgenerational study previously described in^21^. F0 females were exposed to the obesogen TBT via the drinking water. Six F3 and F4 tissues were harvested and processed for DNA methylome and transcriptome analyses. Mesenchymal stem cells (MSCs) were obtained from 8 weeks old F3 and F4 female and male femurs and tibias. Liver and gonadal white adipose tissue (gWAT) were harvested from 33 week old males that had been exposed to a diet challenge. (**B**) Analytical design for the study of the intergenerational and developmental dynamics of TBT-dependent variation of DNA methylomes and transcriptomes. TBT-dependent variation of DNA methylomes and transcriptomes for each tissue was inspected using MBD-seq and RNA-seq, respectively. Names used for each tissue make reference to their generation (F3, F4), sex (F: female, M: Males), and tissue (MSCs, gWAT, and liver). The integrative analysis of TBT-dependent variation of DNA methylomes and transcriptomes for MSCs in F3/F4 females and males would permit inspecting their dynamics through mitosis, meiosis, and epigenetic reprogramming events. The integrative analysis TBT-dependent variation of DNA methylomes and transcriptomes for F4 male MSCs, gWAT and liver would permit inspecting their dynamics through mitosis, and developmental transitions.

Understanding the mechanisms through which effects of environmental exposures can be transmitted to future generations is key to fully comprehend environmental components of human diseases. To further investigate our proposed model of how the putative TBT-dependent disruption of chromatin organization was transmitted through development and across generations, we expanded the integrative analysis of DNA methylome and transcriptome to five additional tissues harvested as part of a transgenerational experiment examining the effect of the ancestral exposure to TBT (Fig. 1)^21^. These tissues included male and female MSCs from F3 and F4 generations and gWAT and liver from F4 males (Fig. 1B). This panel of tissues spans relevant biological variables such as generation (F3 and F4), sex (females and males), ontogeny (endoderm-derived liver vs. mesoderm-derived MSCs and gWAT) and differentiation status (undifferentiated MSCs vs. differentiated adipocytes in gWAT). If TBT caused disruptions of chromatin organization that subsequently biased other genomic traits and were propagated through development and across generations, we would expect that biases in DNA methylation, chromatin accessibility or gene expression should be observed in a broader range of tissues. The gWAT analysis was included here for comparison with our published study^21^. Transgenerational disruption of chromatin organization will be used to refer to this transgenerationally propagated disruption of higher order chromatin organization caused by the ancestral exposure to environmental stressors in the discussion that follows. A key point is that all our biological replicates were from individuals from different litters (i.e., were not siblings or even cousins), thus, they represent bona fide biological replicates. We further note that gWAT, liver and MSCs samples consist of heterogeneous cell populations; therefore, future analyses will be needed to fully understand potential specific effects of TBT on each cell population. This analysis allowed us to assess the dynamics of TBT-dependent variations in chromatin organization through mitosis, meiosis, epigenetic reprogramming and developmental transitions. Therefore, we could make strong inferences on how the putative TBT-dependent disruption of chromatin organization was propagated through development and across generations (Fig. 1B).

Consistent with our previous findings, we observed TBT-dependent disruption of chromatin organization in all tissues under consideration. We inferred that the transgenerational propagation of TBT-dependent disruption of chromatin organization cannot depend exclusively on the faithful replication of altered epigenetic marks, but instead might be able to self-reconstruct in each generation. We also observed similarities between mouse and human genomes with regard to the distribution of genes associated with chromatin organization and metabolic traits and regions of genomic sequence biased toward elevated GC content. This similarity in gene distribution points to the possibility that environmental stressors such as TBT exposure could cause self-reconstructing disruptions in chromatin organization that will predispose both species to metabolic disorders across generations.

## Results

### Tissue dynamics of TBT-dependent variation of DNA methylome and transcriptome

We used methyl-CpG binding domain deep sequencing (MBD-seq) to assess whether a putative TBT-dependent transgenerational disruption of chromatin organization subsequently biased DNA methylation in opposite directions for AT- and GC-enriched genomic regions in our tissue panel. MBD-seq reads for each biological replicate were mapped to consecutive, non-overlapping 100-bp windows for Genome Reference Consortium Mouse build 38 (GRCm38). We used MEDIPS to compare read coverage between TBT and control groups. Windows with significant read coverage differences were denoted as “Differentially Methylated Regions” (DMRs) (Tables S1 and S2 and Fig. S1). DMRs showing higher read coverage in TBT than in control samples were designated as hypermethylated DMRs; DMRs showing lower read coverage in TBT than in control samples were designated as hypomethylated DMRs. The number of DMRs with the same direction of change found in at least two tissues was negligible, even for samples such as F4 male gWAT and liver from the same animal (Table S3). This is hard to reconcile with models proposing that transgenerational phenotypes are dependent on the replication of altered DNA methylation marks through development and across generations. However, they agree well with our proposal that altered DNA methylation marks observed in animals ancestrally exposed to TBT are secondary to a TBT-dependent transgenerational disruption of chromatin organization^21^. We used the concept of isochores to identify concerted biases in DNA methylation variation for five genomic fractions with different GC content. Isochores are large chromosomal regions with a tendency toward base composition uniformity; these are usually categorized in five classes, L1, L2, H1, H2, and H3, from the most AT-enriched to the most GC-enriched^29^. We calculated hyper-/hypomethylated DMR ratios and DMR enrichments per isochore type before and after randomly rearranging data (see Methods for further details; Table S4).

To test whether a putative TBT-dependent transgenerational disruption of chromatin organization subsequently biased gene expression in opposite directions for AT- and GC-enriched genomic regions in our tissue panel, we used RNA-seq to quantify transcript abundance for each biological replicate. RNA-seq reads were mapped to GRCm38 genes and Monte Carlo-Wilcoxon matched-pairs signed-ranks tests (MCW tests) were used to identify concerted biases for the expression of genes located within isochores between TBT and control groups (Table S5 and S6)^21,29–31^. MCW tests calculated gene expression bias indices (GEBIs) before and after randomly rearranging data (see Methods section for further details; Table S6).

**Figure 2:**
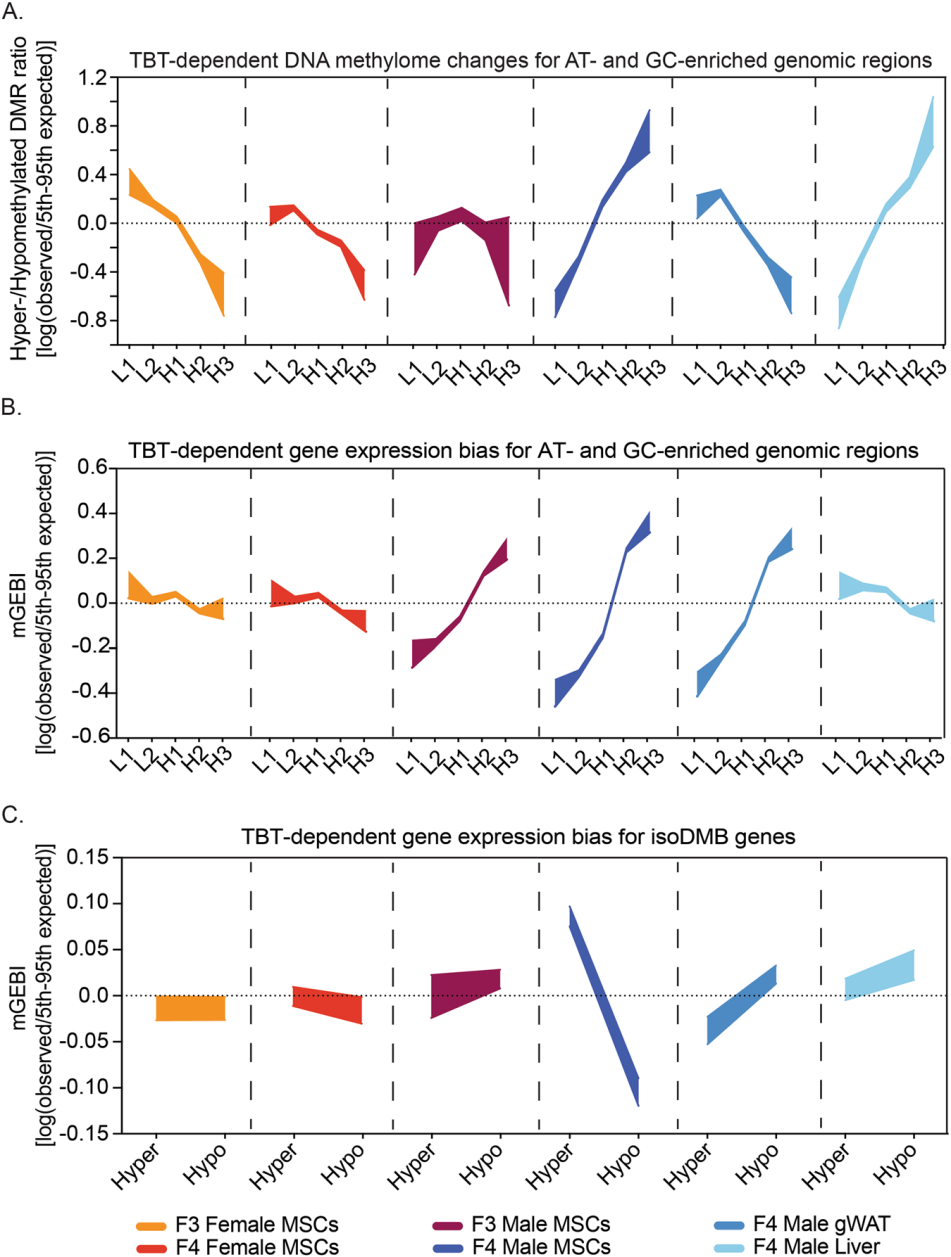
Intergenerational and developmental dynamics of TBT-dependent variation of DNA methylomes and transcriptomes. (**A**) Distribution of TBT-dependent variation for DNA methylomes with regard to regions of the mouse genome defined by their base composition in six somatic tissues. L1, L2, H1, H2 and H3 represent genomic regions with a tendency toward uniformity in base composition or isochores, from the most AT-enriched to the most GC-enriched^29,82^. Hypermethylated or hypomethylated DMRs represent cases for which MBD-seq read coverage was significantly higher or lower in TBT than in control samples, respectively. Hyper-/hypomethylated DMR ratios were calculated as indicated in the Methods section. To assess whether observed hyper-/hypomethylated DMR ratios were significantly different from those expected just by chance, we compared them with hyper-/hypomethylated DMR ratios calculated after randomly rearranging isochore type tags 10,000 times. (**B**,**C**) TBT-dependent biases for the expression of genes located within regions of the mouse genome defined by their base composition or by TBT-dependent variation in DNA methylomes in six somatic tissues. Gene expression bias indices using transcript abundance mean for TBT and control samples (mGEBI) were calculated as indicated in the Methods section. Positive or negative mGEBIs represent cases for which gene expression tends to be higher or lower in TBT than in control samples, respectively. To assess whether observed GEBIs were significantly different from those expected just by chance, we compared them with GEBIs calculated after randomly rearranging signed ranks for each tissue 10,000 times. For each measure and tissue, we draw areas delimited by observed/5th and 95th expected-by-chance percentile ratios. Observed measures were considered significantly higher or lower than measures expected by chance (p < 0.05) if the highlighted area is above or below the 0 value, respectively, and no significantly different from measures expected by chance (p ≥ 0.05) if the highlighted area spanned the 0 value. DMR: differentially methylated region; gWAT: gonadal white adipose tissue; isoDMBs: iso-directional differentially methylated blocks; MSCs: mesenchymal stem cells; TBT: tributyltin.

Analyses of TBT-dependent variation for transcript abundance mean in all tissues, and for DNA methylation in all but one tissue recapitulated the dichotomous distribution we previously reported for TBT-dependent variations in F4 male gWAT DNA methylome and transcriptome and in F3/F4 sperm chromatin accessibility^21^ (Fig. 2A,B, and Tables S4 and S6). For example, comparison of gene expression for MSCs from F4 males ancestrally exposed to TBT and controls revealed that genes located within GC-enriched genomic regions are significantly over-expressed in TBT samples (Fig. 2B). In contrast, genes located within AT-enriched genomic regions are significantly under-expressed in TBT samples (Fig. 2B). The sole exception to the dichotomous pattern concerned the DNA methylome of F3 male MSCs for which both AT-enriched and GC-enriched genomic regions were hypomethylated in TBT samples (Fig. 2A).

Our identification of a generalized dichotomous distribution of TBT-dependent variations in DNA methylation and gene expression linked with regions of biased base composition is consistent with the possibility that ancestral TBT exposure resulted in a disruption of chromatin organization propagated through development and across generations. In turn, such disruption could lead to changes in other genomic traits such as DNA methylation, chromatin accessibility, and gene expression.

### TBT-dependent variations in the DNA methylome and transcriptome are not directly interconnected

The patterns we observed for TBT-dependent variations in DNA methylation and gene expression were not identical for all tissues (Fig. 2A,B, and Tables S4 and S6). However, their differences recapitulate relevant biological variables encompassed by our tissue panel. TBT-dependent alterations observed for the DNA methylomes and transcriptomes of F3 and F4 female and male MSCs showed a distinct sexual dimorphism (i.e. both sexes show alterations and the alterations are different between females and males). When comparing TBT and control samples for each generation, genes located within GC-enriched genomic regions were under-expressed in TBT female samples but over-expressed in TBT male samples. Conversely, genes located within AT-enriched genomic regions were over-expressed in TBT female samples but under-expressed in TBT male samples (Fig. 2A,B, and Tables S4 and S6).

Another instance of differing patterns for TBT-dependent variations in DNA methylome and transcriptome concerns the germ layer of origin. Transcriptome variations for AT- and GC-enriched genomic regions in F4 male MSCs and gWAT (both derived from mesoderm) were similar to each other, but quite different from the endoderm-derived liver (Fig. 2B and Table S6). This is important because variations in a tissue transcriptome are often related to their embryonic origin^32^. Overall, transcriptome/DNA-sequence bias linkage in F4 male tissues reflected both ancestral TBT exposure and the embryologic origin of the tissue. This is consistent with the possibility that the ancestral TBT exposure caused a disruption of chromatin organization that was propagated through development and across generations to bias gene expression, but that the direction of this bias was dependent on other factors such as tissue ontogeny.

In contrast, TBT-dependent variations in DNA methylation in AT- and GC-enriched genomic regions for F4 male tissues do not recapitulate their ontogenetic relationships. Patterns observed in MSCs and liver were similar between themselves, but different from F4 male gWAT (Fig. 2A and Table S4). The DNA methylome is extensively modified during differentiation in general, and adipogenesis in particular^33–36^. Such extensive modification of the DNA methylome could explain why the patterns observed for the TBT-dependent variation of F4 MSCs and gWAT DNA methylomes were so different. Since the liver is composed primarily of differentiated cells such as hepatocytes, it is expected that patterns observed for TBT-dependent variation of F4 liver and gWAT DNA methylomes would be similar to each other, and very different from MSCs. However, DNA methylomes for hepatocytes and undifferentiated cells such as MSCs have been shown to be similar in one regard, they both exhibit accentuated heterogeneities^37–39^. In the case of undifferentiated cells, it has been observed that DNA methylation is very variable among human and murine clonal cells^39^. In the case of hepatocytes, it has been observed that DNA methylation is very variable within and between mouse strains, and even for hepatocytes extracted from single individuals^37,38^. Thus, it is possible that the disparities and similarities we observed for TBT-dependent variations in DNA methylomes across F4 male tissues reflected known DNA methylome dynamics for the major cell types and their differentiated/undifferentiated states encompassed by F4 male MSCs, liver and gWAT. Our results are also consistent with the possibility that a TBT-dependent transgenerational disruption of chromatin organization subsequently biased DNA methylation, and that the direction of biases was dependent on tissue-specific factors. In this case, the direction of observed biases may depend more on the inherent heterogeneity of the DNA methylome for undifferentiated cells and hepatocytes and its extensive modification during adipogenesis rather than tissue ontogeny.

Since TBT-dependent variations of DNA methylomes and transcriptomes reflected different developmental and/or cellular properties encompassed by our tissue panel, we inferred that TBT-dependent alterations for these two traits may not be causally connected (Fig. 2A,B and Tables S4 and S6). We previously used a MCW test scheme to explore possible connections between alterations in DNA methylation and expression of three subsets of genes (Fig. 2C and Table S6)^21^. Subset I comprises genes for which DMRs located close to their transcription start sites might modulate DNA binding of short-range regulators or the basal transcription machinery. Subset II comprises genes for which overlapping or flanking DMRs might modulate binding of DNA topology modifiers, long- and short-regulators, or the basal transcription machinery. Subset III comprises genes located within chromosomal regions punctuated by hypo- or hypermethylated DMRs or isoDMBs. These could reflect regional changes in higher order chromatin structures that subsequently biased genomic traits like DNA methylation and transcription^21^. The only subset for which we detected significant concerted biases in all tissues was subset III, consistent with the possibility that observed changes in DNA methylomes and transcriptomes were both dependent on TBT-dependent regional alterations of chromatin organization (Fig. 2C and Table S6).

Further inspection of the concerted biases observed for the expression of genes located in isoDMBs did not reveal convincing evidence for a direct causal relationship between TBT-dependent variations in DNA methylomes and transcriptomes (Fig. 2C and Table S6). Although the interplay between DNA methylation and transcription is complex^40^, it is widely believed that a major biological function of DNA methylation is to repress gene expression, either directly or together with certain histone modifications^41^. If TBT-dependent variations in DNA methylomes directly drove alterations in gene repression, it would be expected that genes located within hypermethylated isoDMBs were under-expressed in TBT samples, and genes located within hypomethylated isoDMBs were over-expressed. Indeed, as we previously reported, such trends were observed for gWAT in TBT samples (Fig. 2C and Table S6)^21^. However, other tissues did not show the same correspondence between TBT-dependent variations in DNA methylomes and transcriptomes. For instance, genes located within hypomethylated isoDMBs in MSCs from F3, F4 females and F4 males were under-expressed in TBT samples, whereas genes located within hypermethylated isoDMBs in F4 male MSCs were over-expressed in TBT samples (Fig. 2C and Table S6). These results neither fit well with the assumption that the major role of DNA methylation is to repress gene expression, nor do they offer an alternative interpretation for a causal relationship between TBT-dependent variations in DNA methylomes and transcriptomes.

It is known that MBD-seq tends to be more sensitive to highly methylated regions that are CpG enriched, and therefore a better suited method to study DNA methylation variation for GC-enriched regions such as CpG islands and promoters^42^. It could be argued that our analyses of DNA methylation might be misguided by such technical bias of MBD-seq. In fact, consistent with the possibility that MBD-seq was more efficient for the identification of DNA methylation alterations for GC-enriched DNA, we did detect significant enrichments and depletions of TBT-dependent DMRs for GC- and AT-enriched isochores, respectively (Fig. S2 and Table S4). However, the patterns observed for DMR enrichments with regards to AT- and GC-enriched genomic regions are almost identical for the six tissues under study (Fig. S2 and Table S4), and do not recapitulate biologically relevant differences between these tissues like hyper-/hypomethylated DMR ratios do (Fig. 2A and Table S4). Although future confirmations of our analyses will require whole genome bisulfite sequencing, we can discard the hypothesis that these limitations were the sole explanation for the patterns we observed for TBT-dependent variations in DNA methylation across our tissue panel.

In summary, integrative analyses of TBT-dependent variation for DNA methylomes and transcriptomes in our tissue panel suggested that a direct causal relationship between the variations in both traits is unlikely. These results are more consistent with the possibility that biases in DNA methylation and gene expression are both associated with a TBT-dependent transgenerational disruption of chromatin organization and that the direction of biases for each trait was dependent on cell- or tissue-specific properties.

### TBT causes a concerted alteration of the expression of genes related to chromatin organization

Our proposed interpretation of how a TBT-dependent transgenerational disruption of chromatin organization could predispose to obesity offers a possible general mechanism to understand how other chemicals could elicit a similar non-genetic predisposition to metabolic disorders that can be propagated transgenerationally^21^. We found that metabolically-relevant genes were significantly overrepresented in GC-enriched genomic regions. Therefore, disrupted chromatin organization in GC-enriched regions (such as those we inferred from biases in TBT-dependent DNA methylation, transcription, and chromatin accessibility) could result in metabolic disruption that is phenotypically relevant, *per se*, or might be revealed by subsequent dietary or environmental challenges.

**Figure 3:**
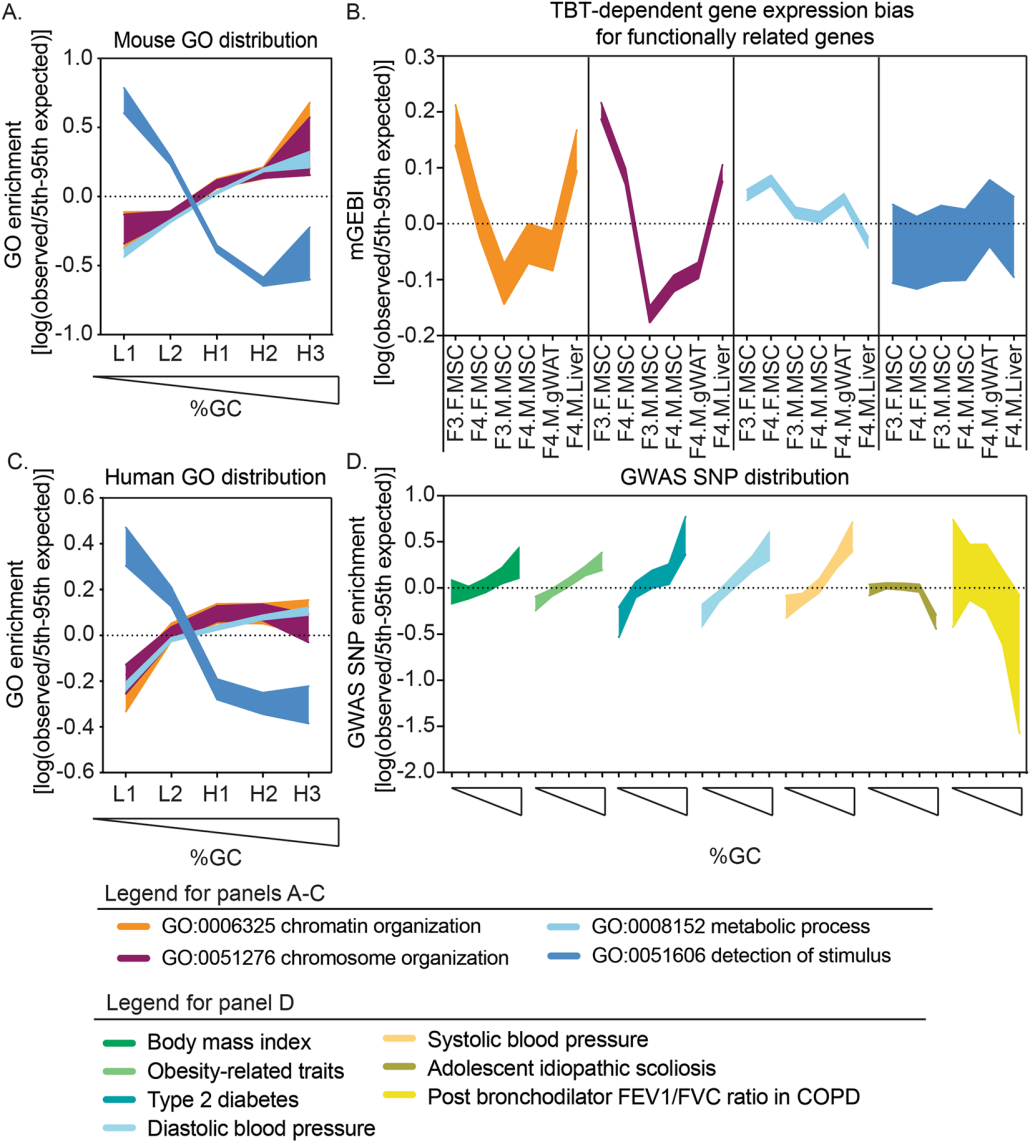
Mouse and human susceptibility to self-propagating disruptions of chromatin organization that predispose to metabolic disorders. (**A**,**C**) Distribution of functionally related genes with regard to AT- and GC-enriched regions of mouse (**A**) and human genomes (**C**). L1, L2, H1, H2 and H3 represent genomic regions with a tendency toward uniformity in base composition or isochores, from the most AT-enriched to the most GC-enriched^29,82^. Mouse and human genes associated with Gene Ontology (GO) terms “*chromosome organization*” (GO:0051276), “*chromatin organization*” (GO:0006325), “*metabolic process*” (GO:0008152), and “*detection of stimulus*” (GO:0051606) were retrieved from the Gene Ontology Consortium database^84^. GO enrichments were calculated as indicated in the Methods section. To assess whether observed GO enrichments were significantly different from those expected by chance, we compared them with GO enrichments calculated after randomly rearranging isochore type tags 10,000 times. (**B**) TBT-dependent biases for the expression of functionally related genes in six somatic tissues. Gene expression bias indices using transcript abundance mean for TBT and control samples (mGEBI) were calculated as indicated in the Methods. Positive or negative mGEBIs represent cases for which gene expression tends to be higher or lower in TBT than in control samples, respectively. indicated in the Methods section. To assess whether observed GWAS SNP enrichments were significantly different from those expected by chance, we compared them with GWAS SNP enrichments calculated after randomly rearranging isochore type tags 10,000 times. For each measure, we draw areas delimited by observed/5th and 95th expected-by-chance percentile ratios. Observed measures were considered significantly higher or lower than measures expected by chance (p < 005) if the highlighted area is above or below the 0 value, respectively, and no significantly different from measures expected by chance (p ≥ 0.05) if the highlighted area spanned the 0 value. F: females; M: males; TBT: tributyltin; MSCs: mesenchymal stem cells; gWAT: gonadal white adipose tissue; NHGRI-EBI: National Human Genome Research Institute-European Bioinformatics Institute; GWAS: genome-wide association studies.

Following the same logic, we asked whether genes encoding proteins that contribute to chromatin organization were also biased toward AT- and GC-enriched genomic regions. If so, their expression could also be susceptible to concerted alterations elicited by a TBT-dependent transgenerational disruption of chromatin organization. To test this hypothesis, we examined the genomic distribution of genes associated with gene ontology (GO) terms referring to different but overlapping levels of chromatin organization: “*chromosome organization*” and “*chromatin organization*”. Consistent with our model, we found that genes associated with both GO terms were significantly overrepresented in GC-enriched regions (Fig. 3A and Table S7). We also examined the distribution of genes associated with GO terms “*metabolic process*” and “*detection of stimulus*”. The location of genes assigned to the “*metabolic process*” term was even more skewed toward GC-enriched regions, whereas those assigned to the “*detection of stimulus*” term were significantly overrepresented in AT-enriched regions (Fig. 3A and Table S7).

To evaluate whether the non-random distributions of genes associated with these GO terms was potentially linked with concerted alteration in their expression induced by TBT-dependent transgenerational disruption of chromatin organization, we performed MCW tests separately for genes associated with “*chromosome organization*”, “*chromatin organization*”, “*metabolic process*”, and “*detection of stimulus*” terms. Genes associated with “*detection of stimulus*” terms, which are overrepresented in AT-enriched regions, showed biased transcript abundance for only one tissue (Fig. 3B and Table S6). Conversely, genes for the other three terms with skewed distributions toward GC-enriched regions showed significantly concerted transcript abundance biases in almost all tissues (Fig. 3B and Table S6).

Remarkably, the direction of the expression biases detected for genes associated with the GO terms “*chromosome organization*” and “*chromatin organization*” recapitulated biological variables encompassed in our tissue panel in the same way as did TBT-dependent variation for the whole transcriptome. MSC patterns showed a reproducible sexual dimorphism, and F4 male patterns recapitulated tissue ontology (Figs 2B and 3B, and Table S6). Transcriptional bias patterns observed for “*metabolic process*” and “*detection of stimulus*” genes did not closely resemble patterns observed for the whole transcriptome (Figs 2B and 3B, and Table S6).

### Humans might be susceptible to environmental disruptions of chromatin organization

The extent to which humans are exposed to TBT and how this may contribute to obesity remains unclear due to a paucity of longitudinal epidemiological studies. Because of their phylogenetic proximity, it is possible that human and mouse genomes share similar organizational principles that made mice susceptible to TBT-dependent transgenerational disruptions of chromatin organization that can self-propagate and predispose direct or ancestrally exposed individuals toward metabolic disorders^21,43^.

To determine whether this is the case, we first examined whether human genes involved with chromatin organization and metabolic processes also showed similar GC-biased genomic location in humans by assessing the distribution of genes with GO terms “*chromosome organization*”, “*chromatin organization*”, “*metabolic process*”, and “*detection of stimulus*”. As in mice, the distribution of genes with the GO term “*detection of stimulus*” were skewed toward AT-enriched genomic regions, and the genomic distribution of “*chromosome organization*”, “*chromatin organization*”, and “*metabolic process*” genes were skewed toward GC-enriched genomic regions (Fig. 3A,C and Table S7).

Secondly, since there are large datasets from human genome-wide association studies (GWAS), it is possible to test whether human genetic variations associated with metabolic diseases or traits are overrepresented in GC-enriched genomic regions as would be expected from the GC-biased location of metabolic genes. Using data from the National Human Genome Research Institute-European Bioinformatics Institute (NHGRI-EBI) GWAS Catalog^44^, we indeed observed that genetic variants associated with five metabolic diseases or traits (“*body-mass index*”, “*obesity-related traits*”, “*type II diabetes*”, “*diastolic blood pressure*”, and “*systolic blood pressure*” were significantly overrepresented in GC-enriched regions of the human genome (Fig. 3D, and Table S8). Genetic variants associated with non-metabolic terms such as “*adolescent idiopathic scoliosis*” and “*post bronchodilator FEV1/FVC ratio in COPD*” did not show similar genomic distributions (Fig. 3D, and Table S8). This argues against the trivial explanation that biased distribution of metabolic genetic variants ultimately echoed the known gene density disparity between AT- and GC-enriched genome regions rather than the biased location of metabolic genes toward GC-enriched genomic regions.

## Discussion

How environmental stressors can predispose exposed individuals and their unexposed descendants to altered phenotype or disease in the absence of DNA mutations is unclear. While a number of well-described transgenerational phenotypes exists^5,6,45,46^, a mechanistic understanding of how effects produced in one generation can be transmitted to others has not been convincingly established. Conceptually, there are two main models to explain how non-genetic alterations produced by environmental stressors could be propagated across generations. These can be termed the replicative model and the reconstructive model^47–49^. The replicative model posits that altered patterns of epigenetic elements causing transgenerational phenotypes are faithfully replicated through development and across generations^47–49^. Considering that epigenetic marks such as DNA methylation or histone modifications undergo intensive reprogramming during development, it is disputed whether the transmission of epigenetic alterations can be explained exclusively, or even predominantly by faithful replication^47–49^. Recent whole genome analyses revealed little or no conservation of altered epigenetic marks between generations and even between somatic cells within the same generation^46,50,51^. The reconstructive model proposes that altered patterns of epigenetic elements causing transgenerational phenotypes are not inherited, but rather recreated in each generation by the priming of altered intermediates^47–49^. There is not much literature on the reconstructive model to date. However, it has been generally proposed that some altered epigenetic marks may escape germline reprogramming events and, together with transmission of ncRNAs in germ cells, cellular signaling or metabolism could prime, in ways yet to be fully understood, the reconstruction of altered epigenetic information in each generation^47–49^.

We studied transgenerational alterations elicited by ancestral TBT exposure for three genomic traits, DNA methylome, transcriptome and chromatin accessibility^21^. Eight tissues were evaluated: F3 and F4 MSCs (female and male), F3 and F4 sperm as well as F4 male gWAT and liver. We observed consistent patterns in all tissues that we interpreted to indicate that ancestral TBT exposure altered chromatin organization and that the altered structures were propagated through development and across generations. We did not observe recurring changes in DNA methylation but did note patterns for TBT-dependent alterations in blocks of DNA methylation and the transcriptome that are largely consistent with important biological variables including sex, tissue ontogeny and differentiation state of cells. Despite the overall consistency of the observed patterns in a broad sense, the specific details are substantially different. This indicates that the propagation of these alterations cannot rely exclusively on faithful replication between tissues and across generations. Rather they are more consistent with a model in which transgenerational propagation of the TBT-dependent predisposition to obesity relies on reconstruction of altered chromatin organizations in each generation. It is unclear whether there are specific altered intermediate changes that prime or facilitate the intergenerational reconstructions of chromatin organization and, if so, what these might be. Potential clues may be found in known chromatin dynamics after fertilization.

Through recent technical innovations, we are starting to appreciate that the spatial organization of eukaryotic chromatin is quite intricate and considerably variable between cell types or even between clonal cells maintained in the same environment^52–55^. Yet, the differentiation of the genome into heterochromatin and euchromatin is persistently recapitulated^29,53,56,57^. Heterochromatin is highly compacted, replicates late in the cell cycle, occupies the nuclear periphery, is AT-enriched and gene-poor, and contains higher levels of non-coding DNA^29^. Euchromatin is relatively uncompacted, replicates early in the cell cycle, occupies the internal area of the nucleus, is GC-enriched, gene-rich and non-coding DNA-poor^29^. Biophysical analyses of eukaryotic nuclei, together with systematic analyses of the spatial proximity of non-juxtaposed loci using chromosome conformation capture methodology revealed that heterochromatic and euchromatic genomic components occupy distinctive nuclear compartments and that compartment boundaries are largely mediated by phase separation^58–61^. Relocation of a locus between euchromatin and heterochromatin revealed that the heterochromatin/euchromatin compartmentalization has multifaceted modulatory effects on replication timing, subnuclear localization, and gene expression^62^. Moreover, disruptions of the heterochromatin/euchromatin phase separation were recently shown to play a key role in the pathogenesis of certain neurodegenerative disorders^63^.

The earliest developmental signs of a complex chromatin organization, including the distinction between heterochromatin and euchromatin, appear during the maternal-to-zygotic (MTZ) transition between oocyte fertilization and the transcriptional activation of the zygotic genome^59,64–67^. During the MTZ transition, maternally-deposited material in the oocyte progressively wanes as zygotic genes become transcriptionally active^64–66^. Processes occurring along the MTZ transition occur at the expense of finite amounts of mostly maternally-deposited material^68,69^. During the MTZ transition, sperm-derived chromosomes undergo intensive chromatin remodeling due to the replacement of protamines by maternally-deposited histones and new chromatin is formed after each zygotic division, which requires substantial amounts of chromatin-forming elements^64–66^.

In principle, variations in genetic and non-genetic determinants of chromatin organization at this critical point in development, when there is no distinction between somatic and germ lines, may prime the early establishment of an altered organization that could then be propagated. The sexual dimorphism many species show for the overall genomic content in heterochromatic repetitive DNA exemplifies how variations in such determinants may modulate the early establishment of chromatin organization. *Y* chromosomes, which are mostly heterochromatic and enriched in repetitive DNA^70^, act as a sink for chromatin-forming elements when such elements are found in limited amounts^71,72^. Thus, whether a sperm nucleus contains an *X* or *Y* chromosome results in a different deployment of chromatin-forming elements throughout the genome in female and in male zygotes. This leads to sexually dimorphic chromatin organization that can subsequently bias transcriptomal, epigenetic, and phenotypic traits^31,73^. Therefore, it could be expected that environmentally-driven quantitative and/or qualitative alterations in chromatin-forming elements deposited in gametes by each parent primed sexually dimorphic alterations in chromatin organization immediately after fertilization. Consequently, sexually dimorphic disruptions in chromatin organization in animals directly or ancestrally exposed to environmental stressors might be a first indication that such stressors could result in alterations of those chromatin-forming elements in gametes needed for the early establishment of chromatin organization just after fertilization.

**Figure 4:**
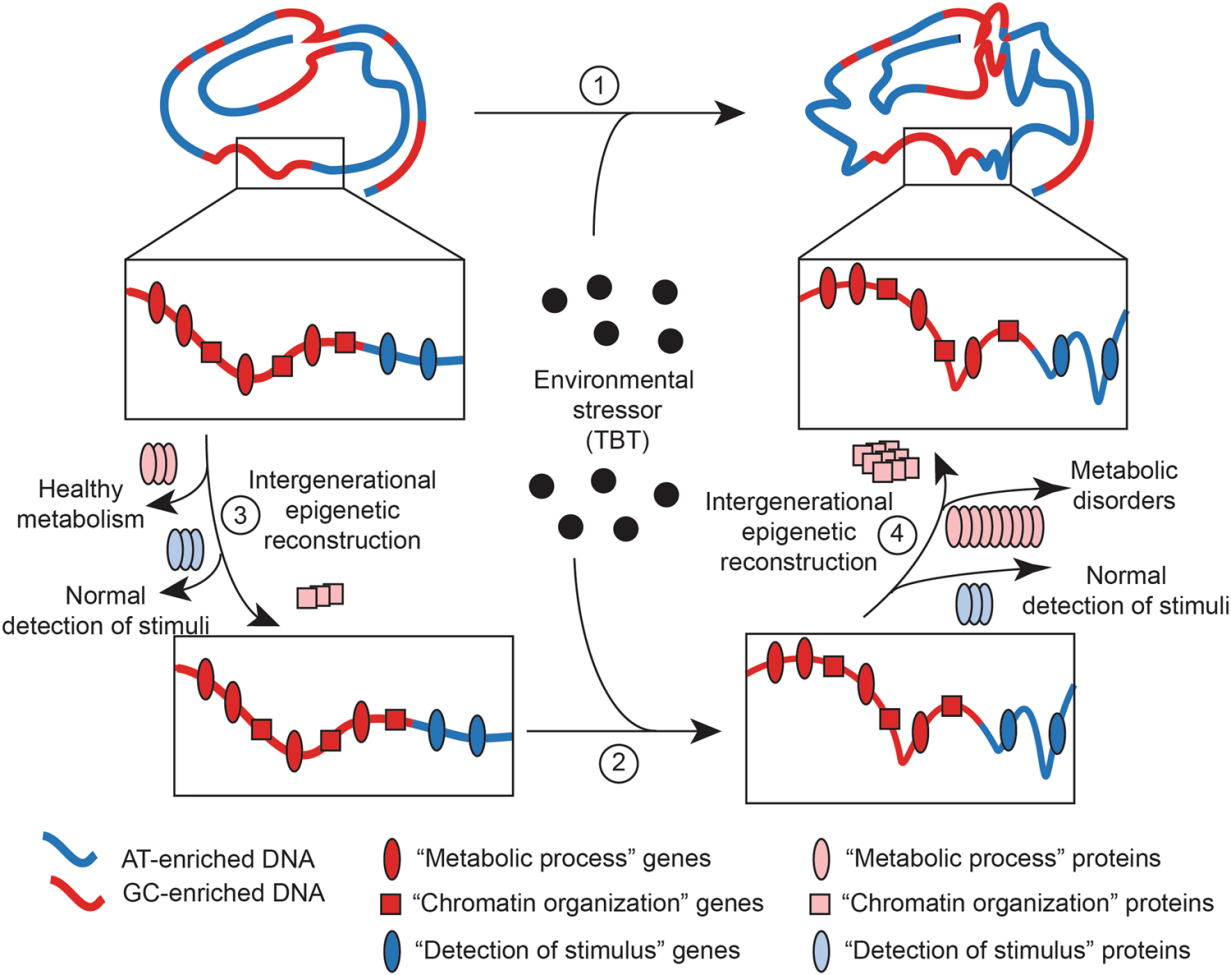
Model for intergenerational reconstructive propagation of environmental disruptions of chromatin organization. Based on our integrative analyses of TBT-dependent variation of DNA methylomes and transcriptomes of F3 and F4, females and males MSCs and F4 male gWAT and liver, and chromatin accessibility of F3 and F4 male sperm, we argue that the exposure to environmental stressors like TBT can cause a self-propagating disruption of chromatin organization, which is symbolized in this cartoon as changes in the spatial arrangement of an ideal chromosome in an ideal tissue (1 and 2). We argue that environmental disruptions of chromatin organization might be able of self-reconstructing through development and across generations and predispose to metabolic disorders because the disruption itself subsequently bias in a concerted way the expression of “chromatin organization” and “metabolic process” genes (3 and 4), which is symbolized in this cartoon as an increase in the number of proteins encoded by each type of genes. Our model does not include which is the nature of the original effect caused by the ancestral exposure to an environmental stressor, e.g., direct disruption of chromatin organization or alteration of chromatin organization determinants (2). MSCs: mesenchymal stem cells; gWAT: gonadal white adipose tissue.

We consistently observed a dichotomous distribution of TBT-dependent variations for DNA methylation, transcription, and chromatin accessibility with regard to AT- and GC-enriched genomic regions in a panel of eight somatic and germline tissues. Because AT- and GC-enriched genomic regions reflect, in part, the heterochromatin/euchromatin organization of eukaryotic genomes^29^, one might expect that ancestral TBT exposure resulted in a disruption of the heterochromatin/euchromatin genomic organization. Consistent with this argument, we observed a distinct sexual dimorphism for the heterochromatin/euchromatin distribution of TBT-dependent alterations of DNA methylomes and transcriptomes in MSCs from F3/F4 females and males. This would be expected if transgenerational propagation of the putative TBT-dependent disruption of the heterochromatin/euchromatin organization resulted from alterations in gamete chromatin organization determinants. Since patterns observed for TBT-dependent variations in gene expression for the entire transcriptome in general and “*chromosome organization*” and “*chromatin organization*” genes in particular recapitulated sex and tissue ontogeny, it is possible that these variations were causally interrelated. One possibility is that TBT-dependent biases observed for the expression of “*chromosome organization*” and “*chromatin organization*” genes were driving the biases observed for the whole transcriptome. This seems unlikely because patterns for TBT-dependent variation for the whole transcriptome in our tissue panel are characterized by opposite directions of change for AT- and GC-enriched genomic regions, and “*chromosome organization*” and “*chromatin organization*” genes are significantly overrepresented only in GC-enriched genomic regions. A feasible alternative is that concerted alterations in the expression of “*chromosome* organization” and “*chromatin organization*” genes resulted in changes at the protein level that then contributed to maintaining and/or propagating the TBT-dependent disruption of chromatin organization. Although the tissues we analyzed did not encompass germ cells, our results support the possibility that TBT-dependent disruptions of chromatin organization were able to self-propagate. This is because the concerted bias in expression of genes that themselves contribute to the establishment, maintenance, and propagation of chromatin organization offers a mechanism for the altered structure to recreate itself in each generation. Thus, TBT-dependent alterations in expression of genes related to chromatin organization in all analyzed tissues is consistent with the possibility that these alterations comprised the altered intermediates that primed the recreation of altered chromatin organization through development and across generations (Fig. 4).

Confirmation that ancestral TBT exposure caused a self-reconstructing disruption of the heterochromatin/euchromatin organization will require new transgenerational experiments. However, observations made in *Drosophila melanogaster* and in humans supports our contention that environmental disruption of chromatin dynamics during early embryogenesis leading to disruption of chromatin organization that subsequently predisposes to metabolic disorders might be common in eukaryotes. First, it is known that the exposure to environmental stressors early in *D*. *melanogaster* embryogenesis results in heterochromatin alterations propagated through development and across generations^74–76^. Second, Öst and coworkers showed that exposing *D*. *melanogaster* males to brief dietary restrictions caused extensive disruptions of the heterochromatin/euchromatin organization in the germ line and their descendants, who also showed a predisposition towards obesity^77^. Third, despite obvious limitations to perform human longitudinal studies spanning analyses of genomic traits in the germ line and/or early embryogenesis, it is noteworthy that in one of the best-studied environmental disruptions of human development, the Dutch Famine Cohort, descendants of women exposed to famine prior to pregnancy and during the first trimester were significantly predisposed to metabolic disorders compared to those exposed later in pregnancy^78^. This is consistent with the possibility that exposure of female gametes to famine contributed to disruptions in chromatin organization very early in embryogenesis.

Our observation of the association between GC-regional bias in chromatin organization and metabolic genes in the mouse and human genomes, together with the similar bias seen in genetic variants associated with human metabolic diseases/traits might indicate that both humans and mice share a genomic organization that could make them susceptible to environmentally-driven disruptions of chromatin organization. This disrupted organization may be able to self-propagate through development and across generations and to predispose naïve descendants of exposed individuals to metabolic disorders. Mice and humans share common principles in their genomic organization, such that environmental disruptions of chromatin organization could be transgenerationally self-reconstructed and phenotypically manifested as metabolic disorders. This could be key for the study of how environmental factors, broadly defined, influence human metabolic disorders. Developmental and intergenerational propagation of environmental disruptions in heterochromatin/euchromatin organization might depend on the reconstruction of extensive genome-wide alterations primed by already altered chromatin organization intermediates. Therefore, it should be expected that detection of such disruptions had escaped analytical approaches that relied primarily on phenotypic variations being caused by a small number of discrete, stable genetic and/or epigenetic alterations^79^. The study of variation in genomic traits with respect to proxies of chromatin organization is sensitive both to the developmental and cellular dynamics of chromatin organization and its disruption following exposure to certain environmental stressors^21,31,77^. Incorporation of chromatin organization-driven analysis of genomic traits to a wider array of samples obtained from animal models and human individuals and/or populations susceptible to environmentally-driven health challenges could be of great benefit in developing analytical tools to better assess environmental components of human disease.

## Materials and Methods

### Experiment description

Procedures using mice were conducted in accordance with Federal guidelines and regulations and were approved by the Institutional Animal Care and Use Committee of the University of California, Irvine (Protocol ID: AUP-17–212). Detailed information about animal procedures were described in a previous publication^21^.

### Tissue collection and sample preparation

Liver and gWAT tissue were isolated from 4 non-sibling, non-cousin randomly selected mice exposed to the diet challenge (33-week-old) and processed as described^21^. MSCs were isolated from femurs and tibias of 8-week-old non-breeding mice. After euthanasia, hind limbs were harvested. Skin, muscle and connective tissue was removed to clean femurs and tibias. Ends of each bone were snipped off with scissors, and warm media (Dulbecco’s modified Eagle medium supplemented with 10% calf bovine serum, 10 mM HEPES, 1 mM sodium pyruvate, 100 IU/mL penicillin, and 100 mg/mL streptomycin) was flushed through the bone marrow cavity with a syringe and a 25 gauge needle. Flushed marrow was collected in a petri dish and broken up by carefully pipetting the media several times. Cell suspensions were strained with a 100 μm nylon strainer to remove cell clumps and bone particles. Cells were centrifuged at 400 × g for 5 min at RT and supernatant was removed. Pellet was resuspended in fresh media, plated in a 10 cm dish and maintained at 37 °C with 5% CO_2_ (passage 0). Media was replaced every 3 days. Cells were trypsinized when they reached 80–90% confluency (6–8 days). MSCs from 3 non-sibling, non-cousin animals from the same treatment group and gender were pooled to generate each of the 5 biological replicates per treatment group. At passage 2, cells were processed for molecular analyses.

### Initial analysis of TBT-dependent variation of DNA methylome and transcriptome

Genomic DNA (gDNA) and RNA were isolated and deep-sequencing downstream analyses performed as described^21^. Raw and processed data for MBD-seq and RNA-seq analyses of F3 and F4 female and male MSCs, and F4 male liver are available at Gene Expression Omnibus (GSE136377). Raw and processed data for MBD-seq and RNA-seq analyses of F4 male gWAT were retrieved from Gene Expression Omnibus (GSE105051). The Supplementary Methods file includes a detailed copy of the R script used here.

We set the threshold of significance for TBT-dependent DNA methylome variation after inspecting the genomic distribution of Differentially Methylated Regions (DMRs) identified using 46 *p* value thresholds ranging between *p* = 0.00001 and *p* = 1 (Supplementary Table S1)^21^. Hypermethylated and hypomethylated DMRs represent cases in which MBD-seq read coverage was significantly higher or lower in TBT than in DMSO samples, respectively. We defined Merged Differentially Methylated Regions (mDMRs) by merging adjacent DMRs with the same direction of change for each tissue and *p* value threshold. We calculated and plot mDMR/DMR ratios for each tissue and *p* value threshold. mDMR/DMR ratio dynamics for each tissue while increasing *p* value thresholds would approximate the independency of newly discovered genomic regions showing significant MBD-seq read coverage. mDMR/DMRs would approach 1 when, upon threshold relaxation, newly identified DMRs tended to be unrelated to already identified DMRs. mDMR/DMRs would approach a minimum (equal to the number of chromosomes/number of genomic 100-bp consecutive, non-overlapping windows for all chromosomes, or 8.07 × 10^−07^) when, upon threshold relaxation, newly identified DMRs tended to be adjacent to already found DMRs. We set the threshold of significance for TBT-dependent methylome changes at *p* = 0.001 (Supplementary Fig. S1 and Table S2). At this point, the mDMR/DMRs ratio consistently decrease for all tissues, meaning that DMRs newly identified using more relaxed thresholds of significance would broaden already identified significant TBT-dependent DNA methylome alterations instead of pointing to new independent ones.

We inspected TBT-dependent transcriptome variation between TBT and DMSO samples using the featureCounts function from Rsubread v1.28^80^ to assign uniquely mapped RNA-seq reads to GRCm38 genes and count reads with the following parameters: GTF.featureType = exon, GTF.attrType = gene_id, allowMultiOverlap = TRUE, nthreads = 24, and strandSpecific = 0. We used cpm and glmQLFTest functions from edgeR v3.20^81^ to estimate the number of counts per million per gene and the statistical significance of RNA-seq reads differential coverage between TBT and DMSO samples.

### Post-analysis of TBT-dependent variations of DNA methylome and transcriptome

Supplementary Tables S2 and S5 provide information for TBT-dependent DNA methylome and transcriptome variation used in the following post-analyses.

We quantified recurrent DMRs (rDMRs) as DMRs with the same direction of change in at least two of the tissues under consideration. No rDMR was found for more than two tissues. For each pair-wise tissue comparisons, we calculated the fraction of rDMRs as A_DMR_ ∩ B_DMR_/A_DMR_ ∪ B_DMR_, where A_DMR_ ∩ B_DMR_ represents the intersection of DMRs for tissues A and B or the number of DMRs found with the same direction of change in both tissues, and A_DMR_ ∪ B_DMR_ represents the union of DMRs for tissues A and B or the number of DMRs found in at least one of the two tissues (Table S3).

We analyzed TBT-dependent DNA methylome and transcriptome variation for our tissue panel with regard to genomic regions with a tendency toward uniformity in base composition or isochores^29^. Isochore coordinates for GRCm38 were retrieved from IsoFinder^82^. Isochores were assorted in types L1, L2, H1, H2, or H3 depending on their GC content as described^29^.

We quantified the number of hypermethylated and hypomethylated DMRs overlapping at least one base of any isochore. For each tissue and isochore type, we calculated hyper-/hypomethylated DMR ratios as (u/d)/(U/D), where u and d represent the number of hypermethylated and hypomethylated DMRs overlapping isochores of each type, respectively, and U and D represent the number of hypermethylated and hypomethylated DMRs overlapping any isochore, respectively (Table S4). For each tissue and isochore type, we calculated DMR enrichments as (x/X)/(l/L), where x and X represent the total number of hypermethylated and hypomethylated DMRs overlapping isochores of each type or any isochore, respectively, and l and L represent the cumulative length of isochores of each type or all isochores, respectively (Table S4). To assess whether observed hyper-/hypomethylated DMR ratios and DMR enrichments were significantly different from those expected just by chance, we compared them with hyper-/hypomethylated DMR ratios calculated after randomly rearranging isochore type tags 10,000 times (Table S4).

To ascertain whether TBT-dependent transcriptome variation tended to be significantly biased in a concerted way for genes sharing relevant traits, we performed Monte Carlo-Wilcoxon matched-pairs signed-ranks tests (MCW)^21,30^. For each tissue under consideration, we selected those genes that were deemed as expressed in both treatments, *i*.*e*., genes for which RNA-seq read coverage was higher than 0 in at least two biological replicates per treatment (Table S5). We defined gene subsets depending on their location with regards to GRCm38 isochores, our previously defined TBT-dependent DMRs (Table S2), or their association with gene ontology (GO) terms (Table S5). GRCm38 genes overlapping isochores, or associated with TBT-dependent DMRs were identified using the tool “Genomic Regions Search” from MouseMine^83^. To account for three possible types of association between TBT-dependent DNA methylome and transcriptome variation, we defined three subsets of genes as described^21^. Subset I includes genes with at least one DMR located less than 1,500 upstream or less than 500 bp downstream of their transcription start site (TSS). Subset II includes genes that overlap or flank at least one DMR. Subset III includes genes located within iso-differentially methylated blocks (isoDMBs), which represent genomic regions punctuated by iso-directional TBT-dependent DMRs. GRC3m8 genes associated with GO terms “*chromosome organization*” (GO:0051276), “*chromatin organization*” (GO:0006325), “*metabolic process*” (GO:0008152), and “*detection of stimulus*” (GO:0051606) were retrieved from the Gene Ontology Consortium database^84^.

MCW test proceeds by comparing gene expression bias indices (GEBI) calculated for the whole transcriptome or a subset of related before and after randomly rearranging data to simulate the effect of chance on the variation in gene expression for a dataset with the same size and expression range^21,30^. We performed MCW tests using transcript abundance mean (M) and coefficient of variation (CV), independently^30^. mGEBI and cvGEBI represent bias indices calculated using transcript abundance mean and CV, respectively. For each subset of genes and tissue, mGEBI was calculated following five steps. First, we subtracted transcript abundance mean for DMSO samples (M_i,DMSO_) from TBT samples (M_i,TBT_) for each gene, *i*.*e*., ∆M_i_ = M_i,TBT_ − M_i,DMSO_. Second, we assigned ranks to all genes in the dataset after sorting them according to the absolute value of ∆M_i_ from lower to higher. Third, we assigned signs to each rank according to the sign of ∆M_i_. Fourth, we sum signed ranks corresponding to genes for the subset under analyses. Fifth, we calculated mGEBIs by dividing the sum of sign ranks for each subset by the maximum value the sum would take if the genes in the subset in question were the ones with highest ∆M_i_ values. Thus, mGEBI for each subset would range from 1 to −1 if the genes in this subset were the ones showing the highest or the lowest ∆M_i_ values, respectively. cvGEBI were calculated in the same way after subtracting for each gene transcript abundance CV for DMSO samples (CV_i,DMSO_) from TBT samples (CV_i,TBT_), *i*.*e*., ∆CV_i_ = CV_i,TBT_ − CV_i,DMSO_. To assess whether observed GEBIs were significantly different from those expected just by chance, we compared them with GEBIs calculated after randomly rearranging signed ranks for each tissue 10,000 times (Table S6).

### Genomic distribution of genes associated with GO terms and genetic variation associated with metabolic diseases/traits

GRCm38 isochore coordinates and overlapping genes were retrieved as described in the previous section. Isochore coordinates for Genome Reference Consortium Human build 37 (GRCh37) were retrieved from IsoFinder^82^. GRCh37 isochore coordinates were converted into Genome Reference Consortium Human build 38 (GRCh38) coordinates using Lift-Over utility from Galaxy v1.0.6^85^. Isochores were assorted in types L1, L2, H1, H2, or H3 depending on their GC content as described^29^. GRCh38 genes overlapping isochores were identified using the tool “Search for features within Genomic Regions” from HumanMine v5.1^86^. GRCh38 genes associated with GO terms “*chromosome organization*” (GO:0051276), “*chromatin organization*” (GO:0006325), “*metabolic process*” (GO:0008152), and “*detection of stimulus*” (GO:0051606) were retrieved from the Gene Ontology Consortium database^84^. For each GO term and isochore type, we calculated GO enrichments as (x/n)/(X/N), where x and X represent the number of genes associated with each GO term overlapping isochores of each type or any isochore, respectively, and n and N represent the number of genes overlapping isochores of each type or any isochore, respectively. To assess whether observed GO enrichments were significantly different from those expected by chance, we compared them with GO enrichments calculated after randomly rearranging isochore type tags 10,000 times (Table S7).

GRCh38 locations for single nucleotide polymorphism (SNPs) associated with diseases/traits “Body mass index”, “Obesity-related traits”, “Type 2 diabetes”, “Diastolic blood pressure”, “Systolic blood pressure”, “Adolescent idiopathic scoliosis”, and “Post bronchodilator FEV1/FVC ratio in COPD” were retrieved from the NHGRI-EBI GWAS Catalog^44^. For each disease/trait and isochore type, we calculated GWAS SNP enrichments as (x/X)/(l/L), where x and X represent the number of SNPs for each trait mapping to isochores of each type or any isochore, respectively, and l and L represent the cumulative length of isochores of each type or all isochores, respectively. To assess whether observed GWAS SNP enrichments were significantly different from those expected by chance, we compared them with GWAS SNP enrichments calculated after randomly rearranging isochore type tags 10,000 times (Table S8).

### Statistical analyses

G*Power v3.1.5 was used to perform a priori power analysis to determine the number of animals required for DNA methylome and transcriptome analyses. Since gene expression changes in tissues are typically ≥ 1.5 fold with SEM of ≤ 10% (effect size d = (μ1−μ2)/σ = 3.92), we set type I and II errors (α and β) at 0.05 and the effect size d = 3.92, the minimum sample size required for a Power (1−β) of 0.95 was calculated to be ≥ 4.

The statistical significance of observed hyper-/hypomethylated DMR ratios, DMR enrichments, GEBIs, GO enrichments, and GWAS SNP enrichments was determined by calculating *p*_*upper*_ and *p*_*lower*_ as the fraction of the 10,000 chance simulations rendering measures that were higher or equal, or lower or equal than observed ones, respectively (Tables S4 and S6–S8). Observed hyper-/hypomethylated DMR ratios, DMR enrichments, GEBIs, GO enrichments, and GWAS SNP enrichments were considered significant if either *p*_*upper*_ or *p*_*lower*_ values were lower than 0.05.

To help comparing visually observed *versus* expected-by-chance contrasts between different traits and tissues, we calculated 5^th^ and 95^th^ percentiles for each 10,000-iteration set of chance simulations, and graphically represented areas limited by the ratios observed/5^th^ and 95^th^ expected-by-chance percentiles using a logarithmic scale. For each trait and tissue under analyses, observed measures were considered significantly higher or lower than measures expected by chance (p < 005) if the highlighted area is above or below the 0 value, respectively, and no significantly different from measures expected by chance (p ≥ 0.05) if the highlighted area spanned the 0 value.

## Supplementary information

Supplementary material

Supplementary Table S1

Supplementary Table S2

Supplementary Table S3

Supplementary Table S4

Supplementary Table S5

Supplementary Table S6

Supplementary Table S7

Supplementary Table S8
