# Supplementary material for: Transgenerational Self-Reconstruction of Disrupted Chromatin Organization After Exposure To An Environmental Stressor in Mice
Source: Sci Rep. 2019 Sep 10;9:13057. doi: 10.1038/s41598-019-49440-2 (PMC6736928; doi:10.1038/s41598-019-49440-2)
Supplement: Supplementary file 1 — Supplementary material [file 41598_2019_49440_MOESM1_ESM.pdf]

TRANSGENERATIONAL SELF-RECONSTRUCTION OF DISRUPTED CHROMATIN ORGANIZATION AFTER EXPOSURE TO AN ENVIRONMENTAL STRESSOR IN MICE

Carlos Diaz-Castillo[1,2,5*], Raquel Chamorro-Garcia[1,2,5*], Toshi Shioda[3*] and Bruce Blumberg[1,4*]

[1]Department of Developmental and Cell Biology, 2011 Biological Sciences 3, University of California, Irvine, 92697-2300, USA.

[2]Current address: Department of Microbiology and Environmental Toxicology, University of California Santa Cruz, CA 95064, USA.

[3]Center for Cancer Research, Massachusetts General Hospital, Bldg 149, 13th Street, Charlestown, MA 02129, USA.

[4]Department of Pharmaceutical Sciences, University of California, Irvine.

[5]These authors contributed equally to this manuscript

*Correspondence to: cdiazcas@ucsc.edu, rchamorr@ucsc.edu, blumberg@uci.edu, shioda@helix.mgh.harvard.edu

SUPPLEMENTARY MATERIAL

Supplementary Methods. Annotated R code for the analysis of TBT-dependent variation of DNA methylomes and transcriptomes for F3 and F4, female and male mesenchymal stem cells (MSCs), and F4 male gonadal white adipose tissue (gWAT) and liver.

Supplementary Figure S1. Independency rate of TBT-dependent variation in DNA methylomes

Supplementary Figure S2. Genomic distribution of TBT-dependent alterations of DNA methylomes.

Supplementary Table S1. Summary of TBT-dependent DNA methylome variation for F3 and F4, female and male mesenchymal stem cells, and F4 male gonadal white adipose tissue and liver using different thresholds of statistical significance.

Supplementary Table S2. Significant TBT-dependent variation of DNA methylomes for F3 and F4, female and male mesenchymal stem cells, and F4 male gonadal white adipose tissue and liver.

Supplementary Table S3. Recurrent TBT-dependent DNA methylome variation for F3 and F4, female and male mesenchymal stem cells, and F4 male gonadal white adipose tissue and liver.

Supplementary Table S4. Distribution of TBT-dependent DNA methylome variation with regard to AT- and GC-enriched genomic regions for F3 and F4, female and male mesenchymal stem cells, and F4 male gonadal white adipose tissue and liver.

Supplementary Table S5. TBT-dependent transcriptome variation for F3 and F4, female and male mesenchymal stem cells, and F4 male gonadal white adipose tissue and liver.

Supplementary Table S6. Results of Monte Carlo-Wilcoxon matched-pairs signed-ranks tests.

Supplementary Table S7. Distribution of groups of genes functionally related with regard to AT- and GC-enriched regions for mouse and human genomes.

Supplementary Table S8. Genomic distribution of genetic variation associated with human diseases or traits with regard to AT- and GC-enriched regions.

SUPPLEMENTARY METHODS

Annotated R code for the analysis of TBT-dependent variation of DNA methylomes and transcriptomes for F3 and F4, female and male mesenchymal stem cells (MSCs), and F4 male gonadal white adipose tissue (gWAT) and liver.

1. DNA methylome analysis

1.1 Requirements: R v3.4, Bioconductor v3.8, MEDIPS v1.30, and BSGenome.Mmusculus.UCSC.mm10 v1.4

1.2 Datasets: F3 female MSCs bam files

f3.f.mscs.DMSO1.meth.merged.sorted.uniq.bam (GSM4047654)

f3.f.mscs.DMSO2.meth.merged.sorted.uniq.bam (GSM4047655)

f3.f.mscs.DMSO3.meth.merged.sorted.uniq.bam (GSM4047656)

f3.f.mscs.DMSO4.meth.merged.sorted.uniq.bam (GSM4047657)

f3.f.mscs.DMSO5.meth.merged.sorted.uniq.bam (GSM4047658)

f3.f.mscs.TBT1.meth.merged.sorted.uniq.bam (GSM4047659)

f3.f.mscs.TBT2.meth.merged.sorted.uniq.bam (GSM4047660)

f3.f.mscs.TBT3.meth.merged.sorted.uniq.bam (GSM4047661)

f3.f.mscs.TBT4.meth.merged.sorted.uniq.bam (GSM4047662)

f3.f.mscs.TBT5.meth.merged.sorted.uniq.bam (GSM4047663)

F3 male MSCs bam files

f3.m.mscs.DMSO1.meth.merged.sorted.uniq.bam (GSM4047664)

f3.m.mscs.DMSO2.meth.merged.sorted.uniq.bam (GSM4047665)

f3.m.mscs.DMSO3.meth.merged.sorted.uniq.bam (GSM4047666)

f3.m.mscs.DMSO4.meth.merged.sorted.uniq.bam (GSM4047667)

f3.m.mscs.DMSO5.meth.merged.sorted.uniq.bam (GSM4047668)

f3.m.mscs.TBT1.meth.merged.sorted.uniq.bam (GSM4047669)

f3.m.mscs.TBT2.meth.merged.sorted.uniq.bam (GSM4047670)

f3.m.mscs.TBT3.meth.merged.sorted.uniq.bam (GSM4047671)

f3.m.mscs.TBT4.meth.merged.sorted.uniq.bam (GSM4047672)

f3.m.mscs.TBT5.meth.merged.sorted.uniq.bam (GSM4047673)

F4 female MSCs bam files

f4.f.mscs.DMSO1.meth.merged.sorted.uniq.bam (GSM4047674)

f4.f.mscs.DMSO2.meth.merged.sorted.uniq.bam (GSM4047675)

f4.f.mscs.DMSO3.meth.merged.sorted.uniq.bam (GSM4047676)

f4.f.mscs.DMSO4.meth.merged.sorted.uniq.bam (GSM4047677)

f4.f.mscs.DMSO5.meth.merged.sorted.uniq.bam (GSM4047678)

f4.f.mscs.TBT1.meth.merged.sorted.uniq.bam (GSM4047679)

f4.f.mscs.TBT2.meth.merged.sorted.uniq.bam (GSM4047680)

f4.f.mscs.TBT3.meth.merged.sorted.uniq.bam (GSM4047681)

f4.f.mscs.TBT4.meth.merged.sorted.uniq.bam (GSM4047682)

f4.f.mscs.TBT5.meth.merged.sorted.uniq.bam (GSM4047683)

F4 male MSCs bam files

f4.m.mscs.DMSO1.meth.merged.sorted.uniq.bam (GSM4047684)

f4.m.mscs.DMSO2.meth.merged.sorted.uniq.bam (GSM4047685)

f4.m.mscs.DMSO3.meth.merged.sorted.uniq.bam (GSM4047686)

f4.m.mscs.DMSO4.meth.merged.sorted.uniq.bam (GSM4047687)

f4.m.mscs.DMSO5.meth.merged.sorted.uniq.bam (GSM4047688)

f4.m.mscs.TBT1.meth.merged.sorted.uniq.bam (GSM4047689)

f4.m.mscs.TBT2.meth.merged.sorted.uniq.bam (GSM4047690)

f4.m.mscs.TBT3.meth.merged.sorted.uniq.bam (GSM4047691)

f4.m.mscs.TBT4.meth.merged.sorted.uniq.bam (GSM4047692)

f4.m.mscs.TBT5.meth.merged.sorted.uniq.bam (GSM4047693)

F4 male liver bam files

f4.m.liv.DMSO1.meth.merged.sorted.uniq.bam (GSM4047694)

f4.m.liv.DMSO2.meth.merged.sorted.uniq.bam (GSM4047695)

f4.m.liv.DMSO3.meth.merged.sorted.uniq.bam (GSM4047696)

f4.m.liv.DMSO4.meth.merged.sorted.uniq.bam (GSM4047697)

f4.m.liv.TBT1.meth.merged.sorted.uniq.bam (GSM4047698)

f4.m.liv.TBT2.meth.merged.sorted.uniq.bam (GSM4047699)

f4.m.liv.TBT3.meth.merged.sorted.uniq.bam (GSM4047700)

f4.m.liv.TBT4.meth.merged.sorted.uniq.bam (GSM4047701)

F4 male gWAT bam files

f4.m.fat.DMSO1.meth.merged.sorted.uniq.bam (GSM2816966)

f4.m.fat.DMSO2.meth.merged.sorted.uniq.bam (GSM2816967)

f4.m.fat.DMSO3.meth.merged.sorted.uniq.bam (GSM2816968)

f4.m.fat.DMSO4.meth.merged.sorted.uniq.bam (GSM2816969)

f4.m.fat.TBT1.meth.merged.sorted.uniq.bam (GSM2816970)

f4.m.fat.TBT2.meth.merged.sorted.uniq.bam (GSM2816971)

f4.m.fat.TBT3.meth.merged.sorted.uniq.bam (GSM2816972)

f4.m.fat.TBT4.meth.merged.sorted.uniq.bam (GSM2816973)

1.3 Setting MEDIPS parameters

```
BSgenome = "BSgenome.Mmusculus.UCSC.mm10"

uniq = 1e-3

extend = 300

shift = 0

ws = 100

selectedchromosomes = c("chr1", "chr2", "chr3", "chr4", "chr5", "chr6", "chr7", "chr8", "chr9", "chr10", "chr11", "chr12", "chr13", "chr14", "chr15", "chr16", "chr17", "chr18", "chr19", "chrX","chrY")


# 1.4 Extracting data from bam files; to run separately for F3 and F4, female and male MSCs and F4 male liver and gWAT bam files


f3.f.mscs.DMSO1.MeDIP <- MEDIPS.createSet(file = "f3.f.mscs.DMSO1.meth.merged.sorted.uniq.bam", BSgenome = BSgenome, extend = extend, shift = shift, uniq = uniq, window_size = ws, chr.select = selectedchromosomes)

f3.f.mscs.DMSO2.MeDIP <- MEDIPS.createSet(file = "f3.f.mscs.DMSO2.meth.merged.sorted.uniq.bam", BSgenome = BSgenome, extend = extend, shift = shift, uniq = uniq, window_size = ws, chr.select = selectedchromosomes)

f3.f.mscs.DMSO3.MeDIP <- MEDIPS.createSet(file = "f3.f.mscs.DMSO3.meth.merged.sorted.uniq.bam", BSgenome = BSgenome, extend = extend, shift = shift, uniq = uniq, window_size = ws, chr.select = selectedchromosomes)

f3.f.mscs.DMSO4.MeDIP <- MEDIPS.createSet(file = "f3.f.mscs.DMSO4.meth.merged.sorted.uniq.bam", BSgenome = BSgenome, extend = extend, shift = shift, uniq = uniq, window_size = ws, chr.select = selectedchromosomes)

f3.f.mscs.DMSO5.MeDIP <- MEDIPS.createSet(file = "f3.f.mscs.DMSO5.meth.merged.sorted.uniq.bam", BSgenome = BSgenome, extend = extend, shift = shift, uniq = uniq, window_size = ws, chr.select = selectedchromosomes)

f3.f.mscs.DMSO.MeDIP <- c(f3.f.mscs.DMSO1.MeDIP, f3.f.mscs.DMSO2.MeDIP, f3.f.mscs.DMSO3.MeDIP, f3.f.mscs.DMSO4.MeDIP, f3.f.mscs.DMSO5.MeDIP)
```

```
f3.f.mscs.TBT1.MeDIP <- MEDIPS.createSet(file = "f3.f.mscs.TBT1.meth.merged.sorted.uniq.bam", BSgenome = BSgenome, extend = extend, shift = shift, uniq = uniq, window_size = ws, chr.select = selectedchromosomes)

f3.f.mscs.TBT2.MeDIP <- MEDIPS.createSet(file = "f3.f.mscs.TBT2.meth.merged.sorted.uniq.bam", BSgenome = BSgenome, extend = extend, shift = shift, uniq = uniq, window_size = ws, chr.select = selectedchromosomes)

f3.f.mscs.TBT3.MeDIP <- MEDIPS.createSet(file = "f3.f.mscs.TBT3.meth.merged.sorted.uniq.bam", BSgenome = BSgenome, extend = extend, shift = shift, uniq = uniq, window_size = ws, chr.select = selectedchromosomes)

f3.f.mscs.TBT4.MeDIP <- MEDIPS.createSet(file = "f3.f.mscs.TBT4.meth.merged.sorted.uniq.bam", BSgenome = BSgenome, extend = extend, shift = shift, uniq = uniq, window_size = ws, chr.select = selectedchromosomes)

f3.f.mscs.TBT5.MeDIP <- MEDIPS.createSet(file = "f3.f.mscs.TBT5.meth.merged.sorted.uniq.bam", BSgenome = BSgenome, extend = extend, shift = shift, uniq = uniq, window_size = ws, chr.select = selectedchromosomes)

f3.f.mscs.TBT.MeDIP <- c(f3.f.mscs.TBT1.MeDIP, f3.f.mscs.TBT2.MeDIP, f3.f.mscs.TBT3.MeDIP, f3.f.mscs.TBT4.MeDIP, f3.f.mscs.TBT5.MeDIP)

# 1.5 Setting a coupling vector

CS <- MEDIPS.couplingVector(pattern = "CG", refObj = f3.f.mscs.DMSO1.MeDIP)

# 1.6 Using edgeR to estimate differential coverage for MBD-seq reads

f3.f.mscs.dmr <- MEDIPS.meth(MSet1 = f3.f.mscs.TBT.MeDIP, MSet2 = f3.f.mscs.DMSO.MeDIP, CSet = CS, p.adj = "fdr", diff.method = "edgeR", MeDIP = FALSE, CNV = FALSE, minRowSum = 10, diffnorm = "quantile")

# 1.7 Setting significance threshold
```

```
p.value <-
c("0.00001","0.00002","0.00003","0.00004","0.00005","0.00006","0.00007","0.00008","0.00009
","0.0001","0.0002","0.0003","0.0004","0.0005","0.0006","0.0007","0.0008","0.0009","0.001","
0.002","0.003","0.004","0.005","0.006","0.007","0.008","0.009","0.01","0.02","0.03","0.04","0.
05","0.06","0.07","0.08","0.09","0.1","0.2","0.3","0.4","0.5","0.6","0.7","0.8","0.9","1")

summary <- data.frame(p.value)

data <- f3.f.mscs.dmr

summary$f3.f.mscs.dmr.hyper <- c(
  sum(data$edgeR.logFC>0 & data$edgeR.p.value<0.00001,na.rm=TRUE),
  sum(data$edgeR.logFC>0 & data$edgeR.p.value<0.00002,na.rm=TRUE),
  sum(data$edgeR.logFC>0 & data$edgeR.p.value<0.00003,na.rm=TRUE),
  sum(data$edgeR.logFC>0 & data$edgeR.p.value<0.00004,na.rm=TRUE),
  sum(data$edgeR.logFC>0 & data$edgeR.p.value<0.00005,na.rm=TRUE),
  sum(data$edgeR.logFC>0 & data$edgeR.p.value<0.00006,na.rm=TRUE),
  sum(data$edgeR.logFC>0 & data$edgeR.p.value<0.00007,na.rm=TRUE),
  sum(data$edgeR.logFC>0 & data$edgeR.p.value<0.00008,na.rm=TRUE),
  sum(data$edgeR.logFC>0 & data$edgeR.p.value<0.00009,na.rm=TRUE),
  sum(data$edgeR.logFC>0 & data$edgeR.p.value<0.0001,na.rm=TRUE),
  sum(data$edgeR.logFC>0 & data$edgeR.p.value<0.0002,na.rm=TRUE),
  sum(data$edgeR.logFC>0 & data$edgeR.p.value<0.0003,na.rm=TRUE),
  sum(data$edgeR.logFC>0 & data$edgeR.p.value<0.0004,na.rm=TRUE),
  sum(data$edgeR.logFC>0 & data$edgeR.p.value<0.0005,na.rm=TRUE),
  sum(data$edgeR.logFC>0 & data$edgeR.p.value<0.0006,na.rm=TRUE),
  sum(data$edgeR.logFC>0 & data$edgeR.p.value<0.0007,na.rm=TRUE),
  sum(data$edgeR.logFC>0 & data$edgeR.p.value<0.0008,na.rm=TRUE),
```

```
sum(data$edgeR.logFC>0 & data$edgeR.p.value<0.0009,na.rm=TRUE),
sum(data$edgeR.logFC>0 & data$edgeR.p.value<0.001,na.rm=TRUE),
sum(data$edgeR.logFC>0 & data$edgeR.p.value<0.002,na.rm=TRUE),
sum(data$edgeR.logFC>0 & data$edgeR.p.value<0.003,na.rm=TRUE),
sum(data$edgeR.logFC>0 & data$edgeR.p.value<0.004,na.rm=TRUE),
sum(data$edgeR.logFC>0 & data$edgeR.p.value<0.005,na.rm=TRUE),
sum(data$edgeR.logFC>0 & data$edgeR.p.value<0.006,na.rm=TRUE),
sum(data$edgeR.logFC>0 & data$edgeR.p.value<0.007,na.rm=TRUE),
sum(data$edgeR.logFC>0 & data$edgeR.p.value<0.008,na.rm=TRUE),
sum(data$edgeR.logFC>0 & data$edgeR.p.value<0.009,na.rm=TRUE),
sum(data$edgeR.logFC>0 & data$edgeR.p.value<0.01,na.rm=TRUE),
sum(data$edgeR.logFC>0 & data$edgeR.p.value<0.02,na.rm=TRUE),
sum(data$edgeR.logFC>0 & data$edgeR.p.value<0.03,na.rm=TRUE),
sum(data$edgeR.logFC>0 & data$edgeR.p.value<0.04,na.rm=TRUE),
sum(data$edgeR.logFC>0 & data$edgeR.p.value<0.05,na.rm=TRUE),
sum(data$edgeR.logFC>0 & data$edgeR.p.value<0.06,na.rm=TRUE),
sum(data$edgeR.logFC>0 & data$edgeR.p.value<0.07,na.rm=TRUE),
sum(data$edgeR.logFC>0 & data$edgeR.p.value<0.08,na.rm=TRUE),
sum(data$edgeR.logFC>0 & data$edgeR.p.value<0.09,na.rm=TRUE),
sum(data$edgeR.logFC>0 & data$edgeR.p.value<0.1,na.rm=TRUE),
sum(data$edgeR.logFC>0 & data$edgeR.p.value<0.2,na.rm=TRUE),
sum(data$edgeR.logFC>0 & data$edgeR.p.value<0.3,na.rm=TRUE),
sum(data$edgeR.logFC>0 & data$edgeR.p.value<0.4,na.rm=TRUE),
sum(data$edgeR.logFC>0 & data$edgeR.p.value<0.5,na.rm=TRUE),
sum(data$edgeR.logFC>0 & data$edgeR.p.value<0.6,na.rm=TRUE),
```

```
  sum(data$edgeR.logFC>0 & data$edgeR.p.value<0.7,na.rm=TRUE),
  sum(data$edgeR.logFC>0 & data$edgeR.p.value<0.8,na.rm=TRUE),
  sum(data$edgeR.logFC>0 & data$edgeR.p.value<0.9,na.rm=TRUE),
  sum(data$edgeR.logFC>0 & data$edgeR.p.value<2,na.rm=TRUE))

summary$f3.f.mscs.dmr.hypo <- c(
  sum(data$edgeR.logFC<0 & data$edgeR.p.value<0.00001,na.rm=TRUE),
  sum(data$edgeR.logFC<0 & data$edgeR.p.value<0.00002,na.rm=TRUE),
  sum(data$edgeR.logFC<0 & data$edgeR.p.value<0.00003,na.rm=TRUE),
  sum(data$edgeR.logFC<0 & data$edgeR.p.value<0.00004,na.rm=TRUE),
  sum(data$edgeR.logFC<0 & data$edgeR.p.value<0.00005,na.rm=TRUE),
  sum(data$edgeR.logFC<0 & data$edgeR.p.value<0.00006,na.rm=TRUE),
  sum(data$edgeR.logFC<0 & data$edgeR.p.value<0.00007,na.rm=TRUE),
  sum(data$edgeR.logFC<0 & data$edgeR.p.value<0.00008,na.rm=TRUE),
  sum(data$edgeR.logFC<0 & data$edgeR.p.value<0.00009,na.rm=TRUE),
  sum(data$edgeR.logFC<0 & data$edgeR.p.value<0.0001,na.rm=TRUE),
  sum(data$edgeR.logFC<0 & data$edgeR.p.value<0.0002,na.rm=TRUE),
  sum(data$edgeR.logFC<0 & data$edgeR.p.value<0.0003,na.rm=TRUE),
  sum(data$edgeR.logFC<0 & data$edgeR.p.value<0.0004,na.rm=TRUE),
  sum(data$edgeR.logFC<0 & data$edgeR.p.value<0.0005,na.rm=TRUE),
  sum(data$edgeR.logFC<0 & data$edgeR.p.value<0.0006,na.rm=TRUE),
  sum(data$edgeR.logFC<0 & data$edgeR.p.value<0.0007,na.rm=TRUE),
  sum(data$edgeR.logFC<0 & data$edgeR.p.value<0.0008,na.rm=TRUE),
  sum(data$edgeR.logFC<0 & data$edgeR.p.value<0.0009,na.rm=TRUE),
  sum(data$edgeR.logFC<0 & data$edgeR.p.value<0.001,na.rm=TRUE),
```

```
sum(data$edgeR.logFC<0 & data$edgeR.p.value<0.002,na.rm=TRUE),
sum(data$edgeR.logFC<0 & data$edgeR.p.value<0.003,na.rm=TRUE),
sum(data$edgeR.logFC<0 & data$edgeR.p.value<0.004,na.rm=TRUE),
sum(data$edgeR.logFC<0 & data$edgeR.p.value<0.005,na.rm=TRUE),
sum(data$edgeR.logFC<0 & data$edgeR.p.value<0.006,na.rm=TRUE),
sum(data$edgeR.logFC<0 & data$edgeR.p.value<0.007,na.rm=TRUE),
sum(data$edgeR.logFC<0 & data$edgeR.p.value<0.008,na.rm=TRUE),
sum(data$edgeR.logFC<0 & data$edgeR.p.value<0.009,na.rm=TRUE),
sum(data$edgeR.logFC<0 & data$edgeR.p.value<0.01,na.rm=TRUE),
sum(data$edgeR.logFC<0 & data$edgeR.p.value<0.02,na.rm=TRUE),
sum(data$edgeR.logFC<0 & data$edgeR.p.value<0.03,na.rm=TRUE),
sum(data$edgeR.logFC<0 & data$edgeR.p.value<0.04,na.rm=TRUE),
sum(data$edgeR.logFC<0 & data$edgeR.p.value<0.05,na.rm=TRUE),
sum(data$edgeR.logFC<0 & data$edgeR.p.value<0.06,na.rm=TRUE),
sum(data$edgeR.logFC<0 & data$edgeR.p.value<0.07,na.rm=TRUE),
sum(data$edgeR.logFC<0 & data$edgeR.p.value<0.08,na.rm=TRUE),
sum(data$edgeR.logFC<0 & data$edgeR.p.value<0.09,na.rm=TRUE),
sum(data$edgeR.logFC<0 & data$edgeR.p.value<0.1,na.rm=TRUE),
sum(data$edgeR.logFC<0 & data$edgeR.p.value<0.2,na.rm=TRUE),
sum(data$edgeR.logFC<0 & data$edgeR.p.value<0.3,na.rm=TRUE),
sum(data$edgeR.logFC<0 & data$edgeR.p.value<0.4,na.rm=TRUE),
sum(data$edgeR.logFC<0 & data$edgeR.p.value<0.5,na.rm=TRUE),
sum(data$edgeR.logFC<0 & data$edgeR.p.value<0.6,na.rm=TRUE),
sum(data$edgeR.logFC<0 & data$edgeR.p.value<0.7,na.rm=TRUE),
sum(data$edgeR.logFC<0 & data$edgeR.p.value<0.8,na.rm=TRUE),
```

```
sum(data$edgeR.logFC<0 & data$edgeR.p.value<0.9,na.rm=TRUE),

sum(data$edgeR.logFC<0 & data$edgeR.p.value<2,na.rm=TRUE))



data.hyper.00001 <- MEDIPS.mergeFrames(frames=subset(data,edgeR.logFC>0 & edgeR.p.value<0.00001))

data.hyper.00002 <- MEDIPS.mergeFrames(frames=subset(data,edgeR.logFC>0 & edgeR.p.value<0.00002))

data.hyper.00003 <- MEDIPS.mergeFrames(frames=subset(data,edgeR.logFC>0 & edgeR.p.value<0.00003))

data.hyper.00004 <- MEDIPS.mergeFrames(frames=subset(data,edgeR.logFC>0 & edgeR.p.value<0.00004))

data.hyper.00005 <- MEDIPS.mergeFrames(frames=subset(data,edgeR.logFC>0 & edgeR.p.value<0.00005))

data.hyper.00006 <- MEDIPS.mergeFrames(frames=subset(data,edgeR.logFC>0 & edgeR.p.value<0.00006))

data.hyper.00007 <- MEDIPS.mergeFrames(frames=subset(data,edgeR.logFC>0 & edgeR.p.value<0.00007))

data.hyper.00008 <- MEDIPS.mergeFrames(frames=subset(data,edgeR.logFC>0 & edgeR.p.value<0.00008))

data.hyper.00009 <- MEDIPS.mergeFrames(frames=subset(data,edgeR.logFC>0 & edgeR.p.value<0.00009))

data.hyper.0001 <- MEDIPS.mergeFrames(frames=subset(data,edgeR.logFC>0 & edgeR.p.value<0.0001))

data.hyper.0002 <- MEDIPS.mergeFrames(frames=subset(data,edgeR.logFC>0 & edgeR.p.value<0.0002))

data.hyper.0003 <- MEDIPS.mergeFrames(frames=subset(data,edgeR.logFC>0 & edgeR.p.value<0.0003))

data.hyper.0004 <- MEDIPS.mergeFrames(frames=subset(data,edgeR.logFC>0 & edgeR.p.value<0.0004))

data.hyper.0005 <- MEDIPS.mergeFrames(frames=subset(data,edgeR.logFC>0 & edgeR.p.value<0.0005))
```

```
data.hyper.0006 <- MEDIPS.mergeFrames(frames=subset(data,edgeR.logFC>0 &
edgeR.p.value<0.0006))

data.hyper.0007 <- MEDIPS.mergeFrames(frames=subset(data,edgeR.logFC>0 &
edgeR.p.value<0.0007))

data.hyper.0008 <- MEDIPS.mergeFrames(frames=subset(data,edgeR.logFC>0 &
edgeR.p.value<0.0008))

data.hyper.0009 <- MEDIPS.mergeFrames(frames=subset(data,edgeR.logFC>0 &
edgeR.p.value<0.0009))

data.hyper.001 <- MEDIPS.mergeFrames(frames=subset(data,edgeR.logFC>0 &
edgeR.p.value<0.001))

data.hyper.002 <- MEDIPS.mergeFrames(frames=subset(data,edgeR.logFC>0 &
edgeR.p.value<0.002))

data.hyper.003 <- MEDIPS.mergeFrames(frames=subset(data,edgeR.logFC>0 &
edgeR.p.value<0.003))

data.hyper.004 <- MEDIPS.mergeFrames(frames=subset(data,edgeR.logFC>0 &
edgeR.p.value<0.004))

data.hyper.005 <- MEDIPS.mergeFrames(frames=subset(data,edgeR.logFC>0 &
edgeR.p.value<0.005))

data.hyper.006 <- MEDIPS.mergeFrames(frames=subset(data,edgeR.logFC>0 &
edgeR.p.value<0.006))

data.hyper.007 <- MEDIPS.mergeFrames(frames=subset(data,edgeR.logFC>0 &
edgeR.p.value<0.007))

data.hyper.008 <- MEDIPS.mergeFrames(frames=subset(data,edgeR.logFC>0 &
edgeR.p.value<0.008))

data.hyper.009 <- MEDIPS.mergeFrames(frames=subset(data,edgeR.logFC>0 &
edgeR.p.value<0.009))

data.hyper.01 <- MEDIPS.mergeFrames(frames=subset(data,edgeR.logFC>0 &
edgeR.p.value<0.01))

data.hyper.02 <- MEDIPS.mergeFrames(frames=subset(data,edgeR.logFC>0 &
edgeR.p.value<0.02))

data.hyper.03 <- MEDIPS.mergeFrames(frames=subset(data,edgeR.logFC>0 &
edgeR.p.value<0.03))
```

```
data.hyper.04 <- MEDIPS.mergeFrames(frames=subset(data,edgeR.logFC>0 & edgeR.p.value<0.04))

data.hyper.05 <- MEDIPS.mergeFrames(frames=subset(data,edgeR.logFC>0 & edgeR.p.value<0.05))

data.hyper.06 <- MEDIPS.mergeFrames(frames=subset(data,edgeR.logFC>0 & edgeR.p.value<0.06))

data.hyper.07 <- MEDIPS.mergeFrames(frames=subset(data,edgeR.logFC>0 & edgeR.p.value<0.07))

data.hyper.08 <- MEDIPS.mergeFrames(frames=subset(data,edgeR.logFC>0 & edgeR.p.value<0.08))

data.hyper.09 <- MEDIPS.mergeFrames(frames=subset(data,edgeR.logFC>0 & edgeR.p.value<0.09))

data.hyper.1 <- MEDIPS.mergeFrames(frames=subset(data,edgeR.logFC>0 & edgeR.p.value<0.1))

data.hyper.2 <- MEDIPS.mergeFrames(frames=subset(data,edgeR.logFC>0 & edgeR.p.value<0.2))

data.hyper.3 <- MEDIPS.mergeFrames(frames=subset(data,edgeR.logFC>0 & edgeR.p.value<0.3))

data.hyper.4 <- MEDIPS.mergeFrames(frames=subset(data,edgeR.logFC>0 & edgeR.p.value<0.4))

data.hyper.5 <- MEDIPS.mergeFrames(frames=subset(data,edgeR.logFC>0 & edgeR.p.value<0.5))

data.hyper.6 <- MEDIPS.mergeFrames(frames=subset(data,edgeR.logFC>0 & edgeR.p.value<0.6))

data.hyper.7 <- MEDIPS.mergeFrames(frames=subset(data,edgeR.logFC>0 & edgeR.p.value<0.7))

data.hyper.8 <- MEDIPS.mergeFrames(frames=subset(data,edgeR.logFC>0 & edgeR.p.value<0.8))

data.hyper.9 <- MEDIPS.mergeFrames(frames=subset(data,edgeR.logFC>0 & edgeR.p.value<0.9))

data.hyper.all <- MEDIPS.mergeFrames(frames=subset(data,edgeR.logFC>0 & edgeR.p.value<2))
```

data.hypo.00001 <- MEDIPS.mergeFrames(frames=subset(data,edgeR.logFC<0 & edgeR.p.value<0.00001))

data.hypo.00002 <- MEDIPS.mergeFrames(frames=subset(data,edgeR.logFC<0 & edgeR.p.value<0.00002))

data.hypo.00003 <- MEDIPS.mergeFrames(frames=subset(data,edgeR.logFC<0 & edgeR.p.value<0.00003))

data.hypo.00004 <- MEDIPS.mergeFrames(frames=subset(data,edgeR.logFC<0 & edgeR.p.value<0.00004))

data.hypo.00005 <- MEDIPS.mergeFrames(frames=subset(data,edgeR.logFC<0 & edgeR.p.value<0.00005))

data.hypo.00006 <- MEDIPS.mergeFrames(frames=subset(data,edgeR.logFC<0 & edgeR.p.value<0.00006))

data.hypo.00007 <- MEDIPS.mergeFrames(frames=subset(data,edgeR.logFC<0 & edgeR.p.value<0.00007))

data.hypo.00008 <- MEDIPS.mergeFrames(frames=subset(data,edgeR.logFC<0 & edgeR.p.value<0.00008))

data.hypo.00009 <- MEDIPS.mergeFrames(frames=subset(data,edgeR.logFC<0 & edgeR.p.value<0.00009))

data.hypo.0001 <- MEDIPS.mergeFrames(frames=subset(data,edgeR.logFC<0 & edgeR.p.value<0.0001))

data.hypo.0002 <- MEDIPS.mergeFrames(frames=subset(data,edgeR.logFC<0 & edgeR.p.value<0.0002))

data.hypo.0003 <- MEDIPS.mergeFrames(frames=subset(data,edgeR.logFC<0 & edgeR.p.value<0.0003))

data.hypo.0004 <- MEDIPS.mergeFrames(frames=subset(data,edgeR.logFC<0 & edgeR.p.value<0.0004))

data.hypo.0005 <- MEDIPS.mergeFrames(frames=subset(data,edgeR.logFC<0 & edgeR.p.value<0.0005))

data.hypo.0006 <- MEDIPS.mergeFrames(frames=subset(data,edgeR.logFC<0 & edgeR.p.value<0.0006))

data.hypo.0007 <- MEDIPS.mergeFrames(frames=subset(data,edgeR.logFC<0 & edgeR.p.value<0.0007))

```
data.hypo.0008 <- MEDIPS.mergeFrames(frames=subset(data,edgeR.logFC<0 & edgeR.p.value<0.0008))

data.hypo.0009 <- MEDIPS.mergeFrames(frames=subset(data,edgeR.logFC<0 & edgeR.p.value<0.0009))

data.hypo.001 <- MEDIPS.mergeFrames(frames=subset(data,edgeR.logFC<0 & edgeR.p.value<0.001))

data.hypo.002 <- MEDIPS.mergeFrames(frames=subset(data,edgeR.logFC<0 & edgeR.p.value<0.002))

data.hypo.003 <- MEDIPS.mergeFrames(frames=subset(data,edgeR.logFC<0 & edgeR.p.value<0.003))

data.hypo.004 <- MEDIPS.mergeFrames(frames=subset(data,edgeR.logFC<0 & edgeR.p.value<0.004))

data.hypo.005 <- MEDIPS.mergeFrames(frames=subset(data,edgeR.logFC<0 & edgeR.p.value<0.005))

data.hypo.006 <- MEDIPS.mergeFrames(frames=subset(data,edgeR.logFC<0 & edgeR.p.value<0.006))

data.hypo.007 <- MEDIPS.mergeFrames(frames=subset(data,edgeR.logFC<0 & edgeR.p.value<0.007))

data.hypo.008 <- MEDIPS.mergeFrames(frames=subset(data,edgeR.logFC<0 & edgeR.p.value<0.008))

data.hypo.009 <- MEDIPS.mergeFrames(frames=subset(data,edgeR.logFC<0 & edgeR.p.value<0.009))

data.hypo.01 <- MEDIPS.mergeFrames(frames=subset(data,edgeR.logFC<0 & edgeR.p.value<0.01))

data.hypo.02 <- MEDIPS.mergeFrames(frames=subset(data,edgeR.logFC<0 & edgeR.p.value<0.02))

data.hypo.03 <- MEDIPS.mergeFrames(frames=subset(data,edgeR.logFC<0 & edgeR.p.value<0.03))

data.hypo.04 <- MEDIPS.mergeFrames(frames=subset(data,edgeR.logFC<0 & edgeR.p.value<0.04))

data.hypo.05 <- MEDIPS.mergeFrames(frames=subset(data,edgeR.logFC<0 & edgeR.p.value<0.05))
```

```
data.hypo.06 <- MEDIPS.mergeFrames(frames=subset(data,edgeR.logFC<0 &
edgeR.p.value<0.06))

data.hypo.07 <- MEDIPS.mergeFrames(frames=subset(data,edgeR.logFC<0 &
edgeR.p.value<0.07))

data.hypo.08 <- MEDIPS.mergeFrames(frames=subset(data,edgeR.logFC<0 &
edgeR.p.value<0.08))

data.hypo.09 <- MEDIPS.mergeFrames(frames=subset(data,edgeR.logFC<0 &
edgeR.p.value<0.09))

data.hypo.1 <- MEDIPS.mergeFrames(frames=subset(data,edgeR.logFC<0 &
edgeR.p.value<0.1))

data.hypo.2 <- MEDIPS.mergeFrames(frames=subset(data,edgeR.logFC<0 &
edgeR.p.value<0.2))

data.hypo.3 <- MEDIPS.mergeFrames(frames=subset(data,edgeR.logFC<0 &
edgeR.p.value<0.3))

data.hypo.4 <- MEDIPS.mergeFrames(frames=subset(data,edgeR.logFC<0 &
edgeR.p.value<0.4))

data.hypo.5 <- MEDIPS.mergeFrames(frames=subset(data,edgeR.logFC<0 &
edgeR.p.value<0.5))

data.hypo.6 <- MEDIPS.mergeFrames(frames=subset(data,edgeR.logFC<0 &
edgeR.p.value<0.6))

data.hypo.7 <- MEDIPS.mergeFrames(frames=subset(data,edgeR.logFC<0 &
edgeR.p.value<0.7))

data.hypo.8 <- MEDIPS.mergeFrames(frames=subset(data,edgeR.logFC<0 &
edgeR.p.value<0.8))

data.hypo.9 <- MEDIPS.mergeFrames(frames=subset(data,edgeR.logFC<0 &
edgeR.p.value<0.9))

data.hypo.all <- MEDIPS.mergeFrames(frames=subset(data,edgeR.logFC<0 &
edgeR.p.value<2))


summary$f3.f.mscs.dmr.hyper.merged <-

  c(nrow(data.up.00001), nrow(data.up.00002), nrow(data.up.00003), nrow(data.up.00004),
nrow(data.up.00005), nrow(data.up.00006), nrow(data.up.00007), nrow(data.up.00008),
nrow(data.up.00009), nrow(data.up.0001), nrow(data.up.0002), nrow(data.up.0003),
```

```
nrow(data.up.0004), nrow(data.up.0005), nrow(data.up.0006), nrow(data.up.0007),
nrow(data.up.0008), nrow(data.up.0009), nrow(data.up.001), nrow(data.up.002),
nrow(data.up.003), nrow(data.up.004), nrow(data.up.005), nrow(data.up.006),
nrow(data.up.007), nrow(data.up.008), nrow(data.up.009), nrow(data.up.01), nrow(data.up.02),
nrow(data.up.03), nrow(data.up.04), nrow(data.up.05), nrow(data.up.06), nrow(data.up.07),
nrow(data.up.08), nrow(data.up.09), nrow(data.up.1), nrow(data.up.2), nrow(data.up.3),
nrow(data.up.4), nrow(data.up.5), nrow(data.up.6), nrow(data.up.7), nrow(data.up.8),
nrow(data.up.9), nrow(data.up.all))

  summary$f3.f.mscs.dmr.hypo.merged <-

    c(nrow(data.down.00001), nrow(data.down.00002), nrow(data.down.00003),
nrow(data.down.00004), nrow(data.down.00005), nrow(data.down.00006),
nrow(data.down.00007), nrow(data.down.00008), nrow(data.down.00009),
nrow(data.down.0001), nrow(data.down.0002), nrow(data.down.0003), nrow(data.down.0004),
nrow(data.down.0005), nrow(data.down.0006), nrow(data.down.0007), nrow(data.down.0008),
nrow(data.down.0009), nrow(data.down.001), nrow(data.down.002), nrow(data.down.003),
nrow(data.down.004), nrow(data.down.005), nrow(data.down.006), nrow(data.down.007),
nrow(data.down.008), nrow(data.down.009), nrow(data.down.01), nrow(data.down.02),
nrow(data.down.03), nrow(data.down.04), nrow(data.down.05), nrow(data.down.06),
nrow(data.down.07), nrow(data.down.08), nrow(data.down.09), nrow(data.down.1),
nrow(data.down.2), nrow(data.down.3), nrow(data.down.4), nrow(data.down.5),
nrow(data.down.6), nrow(data.down.7), nrow(data.down.8), nrow(data.down.9),
nrow(data.down.all))

  significance.threshold.csv <- write.table(summary,file="significance.threshold.csv")

 # 1.8 Extracting DMRs p<0.001

  f3.f.mscs.dmr.001 <- subset (f3.f.mscs.dmr, edgeR.p.value<0.001, select = c(1:3,28,30))

  f3.f.mscs.dmr.001.csv <-
write.table(subset(f3.f.mscs.dmr.001,edgeR.p.value<0.001),file="f3.f.mscs.dmr.001.csv")

# 2. Transcriptome analysis
```

2.1 Requirements: R v3.4, Bioconductor v3.8, Rsubread v1.28, and edgeR v3.20

2.2 Datasets: F3 female MSCs bam files

f3.f.mscs.DMSO1.rna.merged.sorted.bam (GSM4047606)

f3.f.mscs.DMSO2.rna.merged.sorted.bam (GSM4047607)

f3.f.mscs.DMSO3.rna.merged.sorted.bam (GSM4047608)

f3.f.mscs.DMSO4.rna.merged.sorted.bam (GSM4047609)

f3.f.mscs.DMSO5.rna.merged.sorted.bam (GSM4047610)

f3.f.mscs.TBT1.rna.merged.sorted.bam (GSM4047611)

f3.f.mscs.TBT2.rna.merged.sorted.bam (GSM4047612)

f3.f.mscs.TBT3.rna.merged.sorted.bam (GSM4047613)

f3.f.mscs.TBT4.rna.merged.sorted.bam (GSM4047614)

f3.f.mscs.TBT5.rna.merged.sorted.bam (GSM4047615)

F3 male MSCs bam files

f3.m.mscs.DMSO1.rna.merged.sorted.bam (GSM4047616)

f3.m.mscs.DMSO2.rna.merged.sorted.bam (GSM4047617)

f3.m.mscs.DMSO3.rna.merged.sorted.bam (GSM4047618)

f3.m.mscs.DMSO4.rna.merged.sorted.bam (GSM4047619)

f3.m.mscs.DMSO5.rna.merged.sorted.bam (GSM4047620)

f3.m.mscs.TBT1.rna.merged.sorted.bam (GSM4047621)

f3.m.mscs.TBT2.rna.merged.sorted.bam (GSM4047622)

f3.m.mscs.TBT3.rna.merged.sorted.bam (GSM4047623)

f3.m.mscs.TBT4.rna.merged.sorted.bam (GSM4047624)

f3.m.mscs.TBT5.rna.merged.sorted.bam (GSM4047625)

F4 female MSCs bam files

f4.f.mscs.DMSO1.rna.merged.sorted.bam (GSM4047626)

f4.f.mscs.DMSO2.rna.merged.sorted.bam (GSM4047627)

f4.f.mscs.DMSO3.rna.merged.sorted.bam (GSM4047628)

f4.f.mscs.DMSO4.rna.merged.sorted.bam (GSM4047629)

f4.f.mscs.DMSO5.rna.merged.sorted.bam (GSM4047630)

f4.f.mscs.TBT1.rna.merged.sorted.bam (GSM4047631)

f4.f.mscs.TBT2.rna.merged.sorted.bam (GSM4047632)

f4.f.mscs.TBT3.rna.merged.sorted.bam (GSM4047633)

f4.f.mscs.TBT4.rna.merged.sorted.bam (GSM4047634)

f4.f.mscs.TBT5.rna.merged.sorted.bam (GSM4047635)

F4 male MSCs bam files

f4.m.mscs.DMSO1.rna.merged.sorted.bam (GSM4047636)

f4.m.mscs.DMSO2.rna.merged.sorted.bam (GSM4047637)

f4.m.mscs.DMSO3.rna.merged.sorted.bam (GSM4047638)

f4.m.mscs.DMSO4.rna.merged.sorted.bam (GSM4047639)

f4.m.mscs.DMSO5.rna.merged.sorted.bam (GSM4047640)

f4.m.mscs.TBT1.rna.merged.sorted.bam (GSM4047641)

f4.m.mscs.TBT2.rna.merged.sorted.bam (GSM4047642)

f4.m.mscs.TBT3.rna.merged.sorted.bam (GSM4047643)

f4.m.mscs.TBT4.rna.merged.sorted.bam (GSM4047644)

f4.m.mscs.TBT5.rna.merged.sorted.bam (GSM4047645)

F4 male liver bam files

f4.m.liv.DMSO1.rna.merged.sorted.bam (GSM4047646)

f4.m.liv.DMSO2.rna.merged.sorted.bam (GSM4047647)

f4.m.liv.DMSO3.rna.merged.sorted.bam (GSM4047648)

f4.m.liv.DMSO4.rna.merged.sorted.bam (GSM4047649)

f4.m.liv.TBT1.rna.merged.sorted.bam (GSM4047650)

f4.m.liv.TBT2.rna.merged.sorted.bam (GSM4047651)

f4.m.liv.TBT3.rna.merged.sorted.bam (GSM4047652)

f4.m.liv.TBT4.rna.merged.sorted.bam (GSM4047653)

F4 male gWAT bam files

f4.m.fat.DMSO1.rna.merged.sorted.bam (GSM2816958)

f4.m.fat.DMSO2.rna.merged.sorted.bam (GSM2816959)

f4.m.fat.DMSO3.rna.merged.sorted.bam (GSM2816960)

f4.m.fat.DMSO4.rna.merged.sorted.bam (GSM2816961)

f4.m.fat.TBT1.rna.merged.sorted.bam (GSM2816962)

f4.m.fat.TBT2.rna.merged.sorted.bam (GSM2816963)

f4.m.fat.TBT3.rna.merged.sorted.bam (GSM2816964)

f4.m.fat.TBT4.rna.merged.sorted.bam (GSM2816965)

```
# 2.3 Mapping RNA-seq reads to Mus musculus mm10 gene annotation; to run separately for F3 and F4, female and male MSCs and F4 male liver and gWAT bam files

bam.files <- list.files(pattern=".merged.sorted.bam$")

bam.files <- bam.files[-c(2,9)]
```

```
counts <- featureCounts(bam.files, annot.inbuilt = "mm10", isGTFAnnotationFile = FALSE, allowMultiOverlap = TRUE, isPairedEnd = FALSE, nthreads = 24, strandSpecific = 0)

counts.df <- as.data.frame(counts$counts)

colnames(counts.df) <- sub(".merged.sorted.bam", "", colnames(counts.df))

counts.df.f3.f.mscs <- counts.df[, c(1,2,3,4,5,6,7,8,9,10)]

group <- factor(c(1,1,1,1,1,2,2,2,2,2))

design <- model.matrix(~group)


# 2.4 Comparing RNA-seq read coverage for Mus musculus mm10 genes.


f3.f.mscs.dge <- DGEList(counts = counts.df.f3.f.mscs, group = group)

f3.f.mscs.dge <- calcNormFactors(f3.f.mscs.dge)

f3.f.mscs.dge <- estimateDisp(f3.f.mscs.dge, design)

f3.f.mscs.fit <- glmQLFit(f3.f.mscs.dge, design)

f3.f.mscs.qlf <- glmQLFTest(f3.f.mscs.fit, coef = 2)

topTags(f3.f.mscs.qlf)

write.table(cpm(f3.f.mscs.dge), file = "b5.f.mscs.normalized_counts.txt", quote = FALSE, sep = "\t")

write.table(topTags(f3.f.mscs.qlf, n=1000000), file = "f3.f.mscs.DGEsummary.txt", quote = FALSE, sep = "\t")
```

SUPPLEMENTARY FIGURE S1

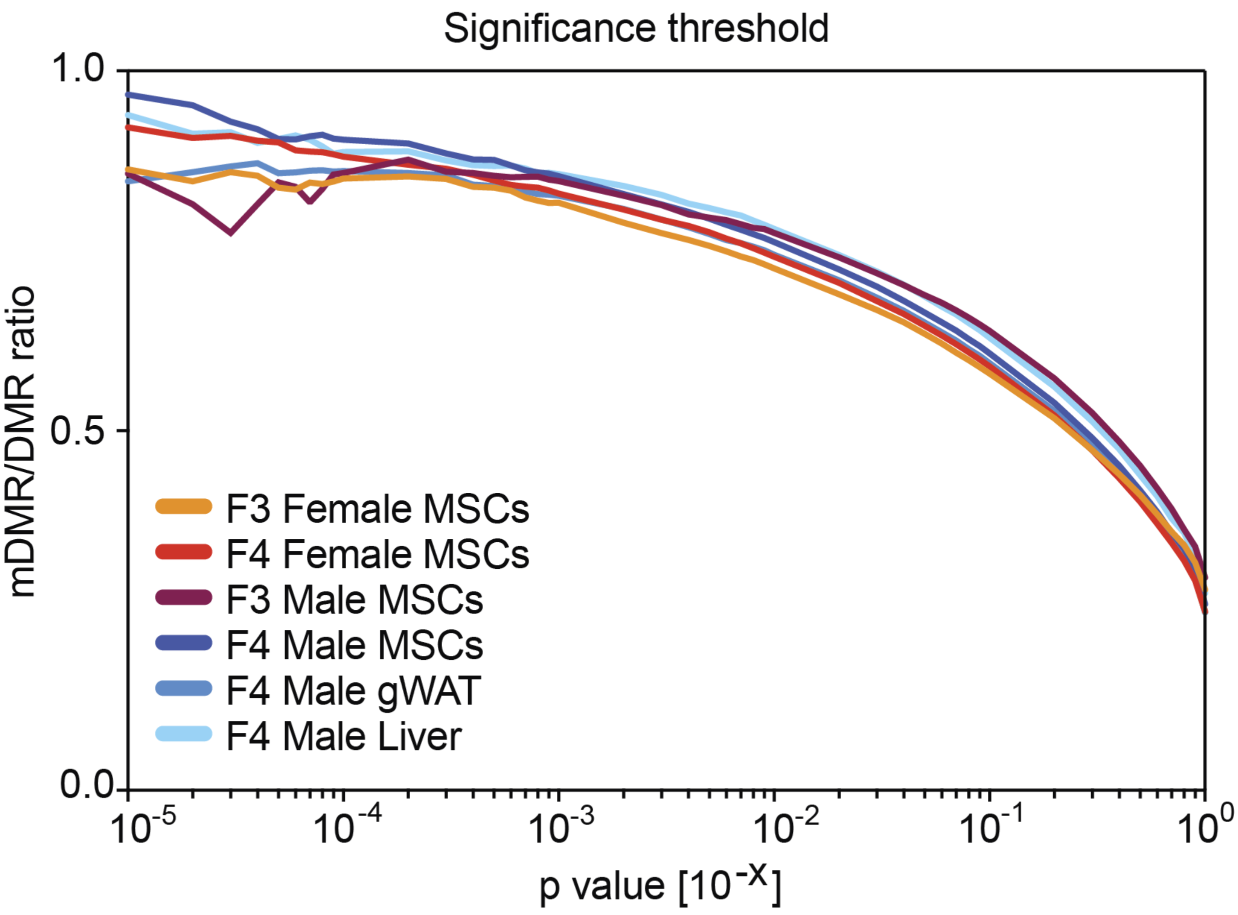

Supplementary Figure S1. Independency rate of TBT-dependent variation in DNA methylomes. Differentially methylated regions (DMRs) refer to 100-bp consecutive, non-overlapping genomic windows showing significant differences in MBD-seq read coverage for TBT and control samples from six somatic tissues defined using MEDIPS. Merged DMRs (mDMRs) result after merging adjacent DMRs with the same direction of change at any given *p* value. The mDMRs/DMRs ratio was used as a measure of new discoveries independency. The mDMRs/DMRs ratio would increase when newly discovered DMRs upon significance relaxation tended to be independent from already identified DMRs, whereas it would decrease when newly discovered DMRs upon significance relaxation tended to be adjacent to already identified DMRs. A threshold of significance for TBT-dependent DMRs was defined at p=0.001, because mDMRs/DMRs ratio clearly decreases for all tissues under consideration. MSCs: mesenchymal stem cells; gWAT: gonadal white adipose tissue.

SUPPLEMENTARY FIGURE S2

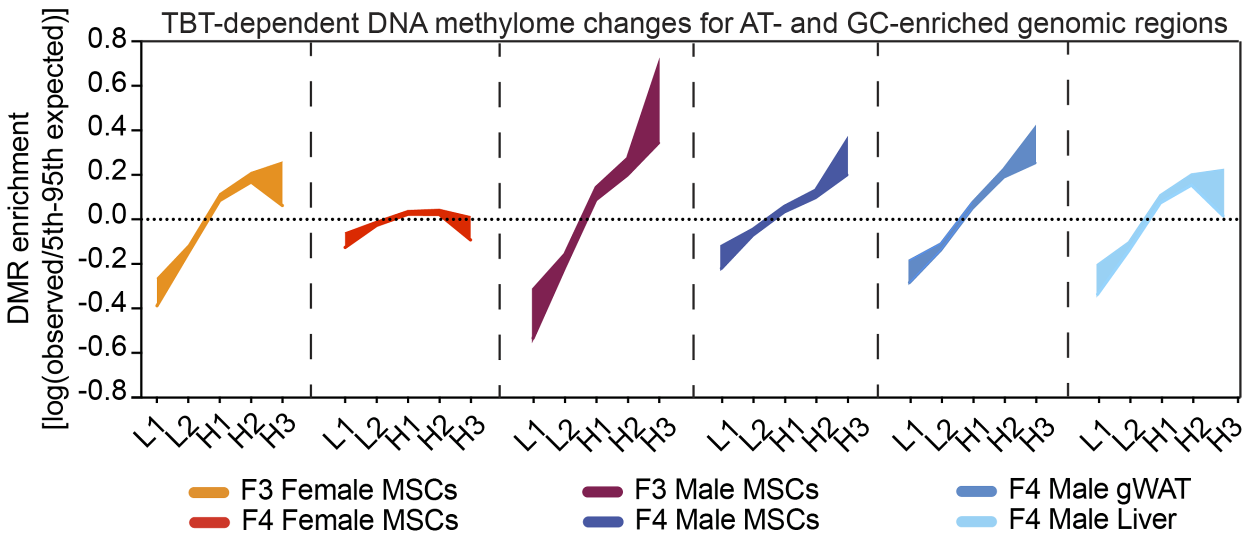

Supplementary Figure S2. Genomic distribution of TBT-dependent alterations of DNA methylomes. Distribution of TBT-dependent variation for DNA methylomes with regard to regions of the mouse genome defined by their base composition in six somatic tissues. L1, L2, H1, H2 and H3 represent genomic regions with a tendency toward uniformity in base composition or isochores, from the most AT-enriched to the most GC-enriched [29,82]. DMR enrichments were calculated as indicated in the Methods section. To assess whether observed DMR enrichments were significantly different from those expected just by chance, we compared them with hyper-/hypomethylated DMR ratios calculated after randomly rearranging isochore type tags 10,000 times. For each measure and tissue, we draw areas delimited by observed/5th and 95th expected-by-chance percentile ratios. Observed DMR enrichments were considered significantly higher or lower than measures expected by chance ($p<0.05$) if the highlighted area is above or below the 0 value, respectively, and no significantly different from measures expected by chance ($p\geq0.05$) if the highlighted area spanned the 0 value. DMR: differentially methylated region; gWAT: gonadal white adipose tissue; MSCs: mesenchymal stem cells; TBT: tributyltin.
